# Basal cell adhesion molecule promotes metastasis‐associated processes in ovarian cancer

**DOI:** 10.1002/ctm2.1176

**Published:** 2023-01-16

**Authors:** Suresh Sivakumar, Sonja Lieber, Damiano Librizzi, Corinna Keber, Leah Sommerfeld, Florian Finkernagel, Katrin Roth, Silke Reinartz, Jörg W. Bartsch, Johannes Graumann, Sabine Müller‐Brüsselbach, Rolf Müller

**Affiliations:** ^1^ Department of Translational Oncology Center for Tumor Biology and Immunology (ZTI) Philipps University Marburg Germany; ^2^ Small Animal Imaging Core Facility Center for Tumor Biology and Immunology (ZTI) Philipps University Marburg Germany; ^3^ Institute for Pathology Philipps University Marburg Germany; ^4^ Bioinformatics Core Facility Center for Tumor Biology and Immunology (ZTI) Philipps University Marburg Germany; ^5^ Cell Imaging Core Facility Center for Tumor Biology and Immunology (ZTI) Philipps University Marburg Germany; ^6^ Clinic for Neurosurgery Philipps University Marburg Germany; ^7^ Biomolecular Mass Spectrometry Max Planck Institute for Heart and Lung Research Bad Nauheim Germany; ^8^ Institute for Translational Proteomics Philipps University Marburg Germany

**Keywords:** ADAM10, BCAM, ovarian cancer, spheroids

## Abstract

**Background:**

Basal cell adhesion molecule (BCAM) is a laminin α5 (LAMA5) binding membrane‐bound protein with a putative role in cancer. Besides full‐length BCAM1, an isoform lacking most of the cytoplasmic domain (BCAM2), and a soluble form (sBCAM) of unknown function are known. In ovarian carcinoma (OC), all BCAM forms are abundant and associated with poor survival, yet BCAM's contribution to peritoneal metastatic spread remains enigmatic.

**Methods:**

Biochemical, omics‐based and real‐time cell assays were employed to identify the source of sBCAM and metastasis‐related functions of different BCAM forms. OC cells, explanted omentum and a mouse model of peritoneal colonisation were used in loss‐ and gain‐of‐function experiments.

**Results:**

We identified ADAM10 as a major BCAM sheddase produced by OC cells and identified proteolytic cleavage sites proximal to the transmembrane domain. Recombinant soluble BCAM inhibited single‐cell adhesion and migration identically to membrane‐bound isoforms, confirming its biological activity in OC. Intriguingly, this seemingly anti‐tumorigenic potential of BCAM contrasts with a novel pro‐metastatic function discovered in the present study. Thus, all queried BCAM forms decreased the compactness of tumour cell spheroids by inhibiting LAMA5 – integrin β1 interactions, promoted spheroid dispersion in a three‐dimensional collagen matrix, induced clearance of mesothelial cells at spheroid attachment sites in vitro and enhanced invasion of spheroids into omental tissue both ex vivo and in vivo.

**Conclusions:**

Membrane‐bound BCAM as well as sBCAM shed by ADAM10 act as decoys rather than signalling receptors to modulate metastasis‐related functions. While BCAM appears to have tumour‐suppressive effects on single cells, it promotes the dispersion of OC cell spheroids by regulating LAMA5‐integrin‐β1‐dependent compaction and thereby facilitating invasion of metastatic target sites. As peritoneal dissemination is majorly mediated by spheroids, these findings offer an explanation for the association of BCAM with a poor clinical outcome of OC, suggesting novel therapeutic options.

## Graphical abstract

Expression of BCAM is associated with a poor survival of ovarian cancer. BCAM reduces compaction of tumour cell spheroids, thereby facilitating their invasion of metastatic sites. Mechanistically, BCAM affects spheroid structure by blocking the interaction of laminin α5 (LAMA5) with integrin β1 (ITGB1). BCAM mediates this function either as a membrane‐bound protein or after shedding (sBCAM) by ADAM10 or 17

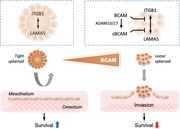

## 1 BACKGROUND

Basal cell adhesion molecule (BCAM) is a member of the immunoglobulin superfamily and is composed of five glycosylated extracellular immunoglobulin domains, a transmembrane domain and a short C‐terminal cytoplasmic tail. BCAM is expressed in two membrane‐bound isoforms of 628 (BCAM1) and 588 amino acids (BCAM2), respectively, that are generated by alternative splicing.^1^ The longer BCAM1 isoform is also known as Lutheran blood group glycoprotein (CD239), as polymorphisms in this gene define the Lu^a^/Lu^b^ Lutheran blood groups.^2^ The shorter BCAM2 isoform lacks a 40‐amino‐acid stretch of the cytoplasmic domain including an SH3‐binding domain with potential signalling function.^3^

BCAM is widely expressed in epithelial, endothelial, hematopoietic and other cell types and has been linked to various human diseases, including gastrointestinal and bladder carcinomas,^4^, ^5^, ^6^, ^7^, ^8^ sickle cell anemia,^9^, ^10^ polycythemia vera^11^ and glomerulonephritis.^12^ BCAM was identified as a receptor for laminins in the extracellular matrix (ECM). It specifically interacts with the laminin α5 (LAMA5) chain,^10^, ^13^, ^14^, ^15^ which, in combination with different β and γ subunits, is a component of laminin‐511 (LN‐511), laminin‐522 and laminin‐523 trimers. In sickle cell disease, BCAM is abundant on erythrocytes, where it mediates the adhesion to LN‐511‐expressing endothelial cells (ECs),^9^, ^10^ and interaction of BCAM on erythroid cells with LAMA5 on ECs has also been observed in polycythemia vera.^11^

Understanding the role of the BCAM–LAMA5 interaction is complicated by the fact that BCAM competes with integrins (α3β1, α6β1, α6β4) for laminin binding,^5^ functionally interacts with the LAMA5 receptor integrin α7β1,^16^ and also functions as a ligand for the α4 subunit of integrin α4/β1 (VLA4) on hematopoietic cells.^17^ It therefore remains unclear whether, or under which conditions, BCAM serves as a signalling receptor, a signalling ligand or a competing molecule.

BCAM is up‐regulated in multiple human tumour entities (www.proteinatlas.org) and has been associated with biological processes linked to tumour progression and metastases, including cell adhesion, motility, migration and invasion. For example, cell adhesion to laminin has been reported to be enhanced following transfection of an unspecified BCAM isoform into 3T3 fibroblasts.^7^ Furthermore, the function of BCAM may depend on the type of cancer, as ectopic expression of BCAM1 in HT1080 osteosarcoma cells decreased adhesion,^5^ while opposite observations were made for motility. Moreover, BCAM‐dependent enhancement of migration was observed in gastric cancer cells,^8^ whereas a decrease was reported following the ectopic expression of BCAM1 into rat hepatoma cells^18^ or MDCK cells, the latter being dependent on the intracellular phosphorylation of serine‐621.^19^ It is likely that these apparent discrepancies are due to context‐ and tumour‐type‐dependent effects. This assumption is supported by the inverse association of *BCAM* expression with overall survival (OS) reported for different tumour entities.^20^

In view of its high expression in ovarian carcinoma (OC; www.proteinatlas.org), the role of BCAM in this cancer entity is of particular interest, yet its function in OC remains enigmatic. A hallmark of OC is its tumour microenvironment (TME), consisting of anatomically and functionally different compartments, that is, the solid (metastatic) tumour masses invading host tissues and the peritoneal fluid, occurring as ascites at advanced stages.^21^, ^22^, ^23^ Due to its pivotal role in peritoneal dissemination, OC ascites differs from the effusions of other human cancers, which are often secondary or reactive. Ascites‐associated cancer cells usually occur as multicellular spheroids, most likely at the root of peritoneal dissemination.^24^

BCAM is also highly abundant as a soluble protein (sBCAM) in OC ascites, and the level of sBCAM is associated with a short relapse‐free survival (RFS).^25^ It is likely that sBCAM is generated by proteolytic cleavage of membrane‐bound BCAM, possibly by matrix metalloproteinases (MMPs), as cleavage of BCAM by MMP14 has been reported.^26^ Consistent with this notion, several members of the MMP family are found in OC ascites, including MMP14.^25^ However, the origin and function of sBCAM in a pathophysiological context have not been addressed to date. Likewise, the role of BCAM in tumour cell spheroids despite their crucial role in OC metastasis remains unknown.

In the present study, we sought (i) to clarify the origins and structure of sBCAM in OC, (ii) to study metastasis‐related functions of the soluble and the two membrane‐bound isoforms of BCAM and (iii) to apply experimental models mimicking the in vivo situation, including tumour cell spheroids, co‐cultures of tumour and mesothelial cells, explanted omentum and a mouse model of peritoneal colonisation.

## 2 METHODS

### 2.1 Patient samples

Ascites and greater omentum tissue with metastatic lesions were collected from patients with ovarian high‐grade serous carcinoma undergoing primary surgery at the University Hospital in Marburg. Patient characteristics are summarised in Tables S1 and S2. Clinical courses were evaluated by RECIST criteria^27^ in patients according to the recommendations by the Gynecologic Cancer InterGroup. Tumour cell spheroids were isolated from ascites as described.^28^, ^29^ All cell populations used for the analysis in this study had a purity of >95%, as determined by flow cytometry and RNA‐sequencing.^28^

### 2.2 Cell cultures

OVCAR4, OVCAR5 and OVCAR8 cells were obtained from the NIGMS Human Genetic Cell Repository of the NIH (Bethesda, Maryland USA). All OVCAR cell lines were cultured in RPMI 1640 (Life Technologies, Darmstadt, Germany) supplemented with 10% FBS (Capricon Scientific, Ebsdorfergrund, Germany). Omentum co‐culture was maintained in DMEM Ham's F‐12 (PAN‐Biotech, Aidenbach, Germany) supplemented with 20% delipidated FBS (Capricon Scientific, Ebsdorfergrund, Germany).

Human peritoneal mesothelial cells were isolated from the omentum of OC patients by a 30‐min digestion of macroscopic tumour‐free omental tissue with trypsin, followed by MACS depletion of contaminating CD45^+^ and EpCAM^+^ cells, as previously described.^30^ Mesothelial cells were cultured in OCMI/5% FCS medium^31^ for a maximum of three to five passages.

### 2.3 Antibodies

Monoclonal anti‐human BCAM antibody (MAB1481 was purchased from R&D Systems/Bio‐Techne (Wiesbaden, Germany); polyclonal anti‐human BCAM antibody (AF148) was purchased from R&D Systems/Bio‐Techne; integrin β1 activating antibody (Ultra‐LEAF™ CD29 antibody; clone TS2/16, #303036) from BioLegend/Biozol (Eching, Germany); integrin β1 blocking antibody (clone 6S6, #MAB2253) from Merck (Darmstadt, Germany); monoclonal anti‐ADAM10 (ab124695) and polyclonal anti‐ADAM17 (ab13535) from Abcam (Berlin, Germany); anti‐CD45‐PE (clone 30‐F11; #553081), anti‐CD45‐APC (clone 30‐F11; #559864), anti‐CD31‐PE (#553373) and anti‐VCAM1/CD106 (clone 429, #561615) from BD BioSciences; anti‐CD45‐AlexaFluor 647 (clone 30‐F11; #103124) from BioLegend. and anti‐CD31‐Vio 667 (clone REA784) from Miltenyi (Bergisch‐Gladbach, Gerany.

### 2.4 Other materials

Recombinant Fc‐BCAM produced in a mouse myeloma cell line (148‐BC), negative control Fc from IgG1 (110‐HG) and marimastat (BB‐2516; #2631) were purchased from R&D Systems/Bio‐Techne and Tocris/Bio‐Techne (Wiesbaden, Germany). Recombinant ADAM10 pro‐domain was kindly provided by Marcia Moss (Verra Therapeutics, Lansing, NY, USA). Recombinant Human Laminin 511 was obtained from BioLamina (LN‐511; Sundbyberg, Sweden) and rat tail collagen I (A1048301) from ThermoFisher Scientific (Dreiech, Germany). N′‐tetrakis(2‐pyridylmethyl)ethylenediamine (TPEN; sc‐200131) was purchased from Santa Cruz (Heidelberg, Germany). Cell Tracker Green CMFDA (#C2925); Cell Tracker Orange CMTMR (#C2927); Cell Tracker blue CMAC (#C2110) and Cell Tracker deep red (#C34565) were from Thermo Fisher (Dreiech, Germany).

### 2.5 Quantification of BCAM by flow cytometry

OVCAR cells were detached from cell culture dishes using Accutase cell dissociation solution (#A6964; Sigma–Aldrich; Taufkirchen, Germany), washed and incubated with monoclonal anti‐human BCAM antibody combined with secondary FITC labelled anti‐mouse IgG (eBioscience/Thermo Fisher Scientific; Dreiech, Germany). Cells were analysed by flow cytometry using a FACS Canto II instrument using Diva Software (BD Biosciences, Heidelberg, Germany). Isotype control antibodies (R&D Systems/Bio‐Techne; Wiesbaden, Germany) were used. Results were calculated as percentage of positive cells and mean fluorescence intensities. Cell death was assessed by propidium iodide staining.

### 2.6 Immunoblotting

Immunoblots were performed according to standard western blotting protocols using the primary antibodies described above in combination with secondary α‐rabbit IgG HRP‐linked polyclonal antibody (Cell Signaling Technology; #7074, RRID:AB_2099233); α‐mouse IgG HRP‐linked polyclonal antibody (Cell Signaling Technology; #7076, RRID: AB_330924) and α‐goat IgG HRP‐linked polyclonal antibody (Jackson ImmunoResearch Labs/Dianova, Hamburg, Germany). Imaging and quantification were carried out using the ChemiDoc MP system and Image Lab software version 5 (Bio‐Rad; Feldkirchen, Germany). For detection of soluble BCAM, confluent cells were cultured in serum‐free medium for 24 h. Conditioned media were collected and concentrated 20‐fold using Vivaspin® 6 centrifugal concentrators (#512‐3777; Sartorius/VWR; Göttingen, Germany) and equal amounts of proteins were mixed with SDS‐PAGE sample buffer and analysed according to standard western blotting protocols. Pierce Reversible Total Protein Staining Kit (Invitrogen/Thermo Fisher Scientific; Dreiech, Germany; #24585) was used for sBCAM immunoblot normalisation.

### 2.7 BCAM ELISA

OVCAR4 cells were seeded in six‐well plates at 5 × 10^5^ cells/well. The confluent cells were washed three times with PBS and serum‐free medium containing either marimastat or ADAM10 prodomain was added. After 24 h, supernatants were collected and soluble BCAM was quantified using human BCAM ELISA (ELH‐BCAM; Ray Biotech/BioCat; Heidelberg, Germany) according to the manufacturer's instructions.

### 2.8 Mass spectrometry of in vitro generated BCAM fragments

One microgram of Fc‐BCAM was incubated with 500 ng of recombinant human ADAM10 (#936‐AD; R&D Systems) in activity buffer (1 mM ZnCl_2_; 20 mM Tris–HCl pH 8,0; 10 mM CaCl_2_; 150 mM NaCl; 0.0006% Brij‐35) for 5 h at 37°C in the presence or absence of the zinc chelator TPEN (50 μM). Samples were prepared for proteomic analysis by acetone precipitation, resolubilisation in 8 M Urea, reduction (10 mM DTT) and alkylation (55 mM iodoacetamide), followed by 2 h incubation with LysC (1:100; Wako Chemicals, Neuss, Germany), dilution to 2 M urea using 50 mM TEAB and overnight digestion using trypsin (1:50; Serva). After solid phase extraction on STAGE tips,^32^ LC/MS^2^‐analysis was performed as described^29^ and data analysed with MaxQuant using the human Uniprot database (canonical and isoforms;194237 entries; downloaded 2021/02/08). The relevant instrument as well as MaxQuant parameters are extracted using MARMoSET and included in the supplementary material.

### 2.9 Protein mass spectrometry (MS) of BCAM in tumour‐cell‐conditioned media

For proteomic analyses of conditioned media an earlier dataset of ascites‐derived tumour cells^29^ from ovarian high‐grade serous carcinoma patients was researched using a semi‐specific MaxQuant search^33^, ^34^ against the Uniprot human database (canonical and isoforms;1888349 entries; downloaded 2020/02/05). Instrument parameters used were extracted using MARMoSET^35^ and are together with relevant MaxQuant parameters included in Supplemental Methods S1 and S2.

### 2.10 siRNA‐mediated interference

siRNA transfection was performed in OVCAR4 cells cultured in RPMI plus 10% FCS using the Lipofectamine 2000 (Invitrogen/Thermo Fisher Scientific; Dreiech, Germany; #11668027) reagent according to the manufacturer's protocol. BCAM, ADAM10 and ADAM17 siRNA‐mediated interference was performed using three different siRNA oligonucleotides (Sigma–Aldrich; Taufkirchen, Germany): BCAM #1 (5′‐GAGACUACGUGUGCGUGGU‐3′), BCAM #2 (5′‐GGAU UACGACGCGGCAGAU‐3′), BCAM #3 (5′‐ CAGAGCUAAAGACAGCGGA ‐3′);ADAM10 #1 (5′‐CAGUCAUGUUAAAGCGAUU‐3′), ADAM10 #2 (5′‐GAACUAUGGGUCUCAUGUA‐3′), ADAM10 #3 (5′‐CGCAUAAGAAUCAAUACAA‐3′);ADAM17 #1 (5′‐CAUCAAGUACUGAACGUUU‐3′), ADAM17 #2 (5′‐CUUAGCAGAUGCUGGUCAU‐3′), ADAM17 #3 (5′‐CAAUCUAUAAGACCAUUGA‐3′). MISSION siRNA Universal Negative Control #1 from Sigma–Aldrich (Taufkirchen, Germany) was used as a control. Cells were harvested 72 h after transfection.

### 2.11 Stable BCAM overexpression in OVCAR8 cells

BCAM overexpression was achieved by transient transfection of OVCAR8 cells with BCAM1 vector (Transcript variant 1; OHu20355 – Gencript Biotech; Piscataway, NJ) or BCAM2 vector (Transcript variant 2; OHu07730 – Gencript Biotech, Piscataway, NJ) or empty pCDNA3.1 control (GenScript Biotech, Piscataway, NJ) using TransIT‐X2 (MirusBio/Mobitec; Göttingen, Germany) according to the manufacturer's instructions. Cells were selected in the presence of Geneticin (G418) (Santa Cruz, Heidelberg, Germany; #sc‐29065B) (0.8 mg/ml) and stable clones were analysed for BCAM expression by RT‐qPCR, immunoblotting and FACS analysis using the following PCR primers (Sigma‐Aldrich; Taufkirchen, Germany): 5′‐ TGCGCGTGGCCTATCTGGAC (forward) and 3′‐ CTTGGTCCAGCGTAGGGCAGG (reverse).

### 2.12 CRISPR/Cas9‐mediated BCAM disruption OVCAR8 cells

The pX330‐U6‐Chimeric_BB‐CBh‐hSpCas9 vector with puromycin selection marker was a gift from Dr. Elke Pogge von Strandmann. sgRNAs targeting BCAM were cloned into linearised px330 vector. The sequences used for sgRNA are as follows:

sgRNA #1 – (5′‐ CACCTGCGAGCAACAGCAGCCGCG‐3′),

sgRNA #2 – (5′‐CACCACTGCGAGCAACAGCAGCCG‐3′),

sgRNA #3 – (5′‐ CACCGCGCTTGTCTGTACCCCCGCTGG‐3′).

After transfection using TransIT‐X2 the OVCAR8 cells were placed into 96‐well plates at the concentration of 1 cell/well. Single colonies were picked, and gene disruption was validated by immunoblot analysis. Control clones were generated by transfection of the empty px330 vector.

### 2.13 xCELLigence real‐time cell analysis of tumour cell adhesion and migration

Adhesion and migration assays were performed using the xCELLigence real‐time cell analysis (RTCA) DP instrument (ACEA Biosciences/Agilent; Waldbronn, Gemany) placed in a humidified incubator at 37 °C and 5% CO_2_. For cell adhesion assays, E‐plates‐16 (ACEA Biosciences/Agilent; Waldbronn, Germany; #2801032) were used. The plates were coated with LN‐511 or COL1 overnight at 4°C, washed with PBS and blocked with 1% bovine serum albumin in PBS for 1 h at 37°C. After blocking, the wells were washed, and 20,000 cells detached with Accutase solution were seeded per well. Impedance was measured every 3 min for the first 8 h, followed by every 15 min for the next 12 h. The cell index indicates the microelectrode impedance, which corresponds to the strength of cell adhesion.

For cell migration assays, CIM‐16 plates (ACEA Biosciences/Agilent; Waldbronn, Germany; #2801038) were used. These plates resemble Boyden chamber plates with upper and lower compartments separated by an 8 μm pore‐containing membrane, with the impedance‐measuring electrodes at the lower side. Following coating with LN‐511 or COL1 overnight at 4°C, plates were washed and blocked with 1% bovine serum albumin in PBS for 1 h at 37°C. Wells were rinsed with PBS and 60,000 cells in serum‐free medium were seeded into the upper compartment and allowed to migrate to the lower compartment containing medium with 10% serum. Impedance was measured every 15 min for 48 h. The cell index corresponds to the number of cells that migrated through the pores.

### 2.14 Tumour cell motility

For cell motility, untreated μ slide VI uncoated 0.1 polymer coverslips (Ibidi; Gräfelfing, Germany) were coated with human recombinant laminin‐511 and blocked with 1% BSA. OVCAR cells were labelled with cell tracker green CMFDA) according to the manufacturer's protocol and detached with Accutase solution. Labelled cells were seeded at 20,000 cells/30 μl channel and grown for 3 h in complete medium. Cells were then incubated at 37°C in serum‐free medium for 1 h in a CO_2_ microscope stage incubator before monitoring cell migration by spinning‐disc microscopy without medium change. Fluorescence images (488 nm laser) were taken at 10‐min intervals for 12 h at 37°C and 5% CO_2_ using an AxioObserver Z1 microscope and AxioVision software (Carl Zeiss). Images were processed with Imaris software (Oxford instruments; Wiesbaden, Germany), and the positions of cells were tracked using Imaris 3D tracking algorithm (Brownian motion) to quantify cell motility. For each condition, cells were tracked from 5 different fields. Trajectory plot combined with motility rate (μm/h) was plotted using Python functions.

### 2.15 Spheroid formation and analysis

Spheroids were formed by seeding 2500 cells in a 96‐well U‐bottom cell‐repellent plate (Greiner Bio‐One; Frickenhausen, Germany; #650970). For the experiments in Figure 5, spheroids were formed in the presence of 20 μg/ml control IgG1, integrin β1 activating antibody, 10 μg/ml integrin β1 blocking antibody or 10 μg/ml LN‐511. Cells were monitored with a DMI3000B microscope (Leica; Wetzlar, Germany). Images were processed using ImageJ/Fiji software. Circularity was calculated using an inbuilt feature of ImageJ software. The percentage of gaps as a reflection of spheroid compactness was calculated as (gap area of spheroid/ total area of spheroid) ×100.

### 2.16 Spheroid dispersion in a 3D collagen matrix

Spheroids were formed as described above. Collagen I was neutralised 1 N NaOH, adjusted to a final concentration of 2 mg/ml, added to 96‐well plates (50 μl/well) and incubated for 5 min at room temperature. One spheroid was transferred to each well, incubated with 75 μl of COL1 solution for 1 h at 37°C and overlaid with 100 μl of medium. Spheroids were monitored and pictures were taken using Leica SP8i confocal microscopy (Leica; Wetzlar, Germany). Images were processed using ImageJ/Fiji software for further quantification. Percent dispersion was calculated as (area at 48 h − area at 0 h)/area at 0 h × 100.

### 2.17 Mesothelial cell clearance

Spheroids were generated as described above using tumour cells labelled with Cell Tracker Green. 96‐well plates were coated with collagen I at a concentration of 5 μg/cm^2^ for 45 min at 37°C. Mesothelial cells labelled with Cell Tracker Orange were seeded in 96‐well plates (14,000 cells/well) and allowed to form a confluent monolayer. After 4 days, 1 spheroid was transferred to each well and monitored for 48 h by Leica SP8i confocal microscopy (Leica; Wetzlar, Germany). Images were processed using ImageJ/Fiji software for further quantification. Mesothelial cell clearance was calculated as cleared area at 48 h/spheroid area at 0 h.

### 2.18 Immunohistochemistry of BCAM, LAMA5, COL1 and COL4

For immunohistochemistry, heat‐induced epitope retrieval was performed with EDTA for LN‐511 subunit LAMA5, COL1 and COL4 and with Trilogy for BCAM. Staining was performed on a DAKO autostainer plus. After blocking endogenous peroxidase, sections were incubated for 45 min with mouse monoclonal anti‐BCAM antibody (1:25; R&D systems #MAB1481, clone 87207), mouse monoclonal anti‐LAMA5 antibody (1:50; Atlas Antibodies # AMAb91124, clone CL3118), mouse monoclonal anti‐Collagen IV antibody (1:100; Dako/Agilent; Waldbronn, Germany; #M0785, clone CIV22) or rabbit polyclonal anti‐Collagen I antibody (1:200; Abcam #ab34710). Sections were washed and incubated with Dako REAL EnVision HRP Rabbit/Mouse polymer, which reacts with DAB‐Chromogen, according to the manufacturer´s protocol.

### 2.19 Omentum model

Spheroids were generated by seeding 250,000 pre‐labelled cells (0.5 μM Cell Tracker Green) in a 24‐well ultra‐low attachment plate (Merck; Darmstadt, Germany; #CLS3473‐24EA). After 72 h spheroids of two wells were collected for each omentum. Mice were maintained and handled according to the internal approval by the local animal welfare officers. The protocol of Khan et al.^36^ was applied with some modifications. C57Bl/6 J mice (8‐12 weeks old; Charles River Laboratories, Sulzfeld, Germany) were sacrificed and the omentum, pancreas and spleen were excised *en bloc* by cutting the connections to the gastrointestinal tract and placed in ice‐cold PBS, where pancreas and spleen remained at the bottom, while the adipose‐rich omentum floated on the surface. By trimming its base, the omentum was separated from the surrounding organs. The omentum was placed in a reaction tube containing 3 ml of tumour cell spheroid suspension corresponding to 5 × 10^5^ cells and kept under rotation at 37°C, 2% O_2_, 93% N_2_ and 5% CO_2_ for 3 h. The spheroid suspension was then replaced by culture medium and the tissue was kept under rotation for an additional hour to remove loosely and non‐attached cells.

Next, the omentum was attached to a Millicell culture insert (Merck Millipore, #PIC03050) using Cell‐Tak Cell and Tissue Adhesive (Corning/VWR, Göttingen, Germany; #10317081). For this purpose, 7.5 μl Cell‐Tak was spread evenly on the insert membrane and air‐dried under laminar flow at room temperature. After two washing steps with 1 ml sterile water, the membrane was air‐dried and the insert was placed in a 6‐well culture plate. To allow optimal attachment, the omentum was placed on the pre‐coated insert membrane without medium for 1 min. Three millilitres of culture media were pipetted into the insert and 2 ml into the surrounding well. Co‐culture was carried out under hypoxic conditions^37^ for additional 44 h at 37°C and 2% O_2_, 93% N_2_ and 5% CO_2_. Under these conditions, a similar intensity of VCAM1 immunostaining was observed during the observation period of the experiments, indicating that no significant activation or senescence occurred during the chosen incubation period (see *Results* section for details).^38^

### 2.20 Whole mount staining and fluorescence microscopy

After co‐culture with tumour cells, whole mount staining of the omentum was performed based on a published protocol^39^ as follows. The omentum was transferred into a 5 ml reaction tube, blocked using TruStain FcX (BioLegend; Amsterdam, Netherlands; #101320) at 10 μg/ml in 200 μl of PBA (PBS, 1% BSA and 0.1% sodium azide) at 4°C under rotation for 10 min. Antibodies were then added directly to the tube and incubated for 2 h at 4°C under rotation (anti‐CD45‐PE‐Vio615 6 μg/ml; anti‐CD31‐PE: 10 μg/ml; anti‐CD45‐AlexaFluor 647: 12.5 μg/ml; anti‐CD45 APC 5 μg/ml). After washing in 2 ml PBA at 4°C under rotation the tissue was embedded in 1% agarose and fixed in a glass cuvette filled with PBS.

Fluorescence microscopy was performed on a Luxendo LCS SPIM light‐sheet Microscope (Bruker Corp.; Billerica, MA, USA) using the following laser and filter settings: 488 nm with BP 500–530; 561 nm with BP 580–627; 642 nm with BP 655–704. Excitation and detection were performed via 4× objectives (excitation: Nicon, numerical aperture 0.13; detection: Olympus, numerical aperture 0.28) and images were taken in 2.2‐fold magnification.

We also performed multiphoton microscopy to visualise collagen fibres by second‐harmonic generation on an FVMPE‐RS Multiphoton Microscope (Olympus; Hamburg, Germany) equipped with Spectra Physics pulsed laser Insight DeepSee 690 – 1300 nm and MaiTai Sa 690–1040 nm. A 25‐fold magnification was achieved with a water immersion objective with a numerical aperture of 1.05. Z‐stack images were taken from different regions of the omentum. For this purpose, the tissue was fixed on a cover slip (NeoLab; Heidelberg, Germany; #1‐6292) by covering with 1% agarose in PBS. Image analysis was performed with Imaris software 9.9.0 (Bitplane) where fluorescently labelled tumour cells were defined and counted as spots or surfaces.

### 2.21 Quantification of OVCAR8 invasion into mouse omentum by Taqman‐PCR

Genomic DNA was isolated from the omentum using the NucleoSpin DNA lipid tissue kit (Macherey‐Nagel; Düren, Germany; Cat# 740471.50) according to the manufacturer's instructions. Tissue lysis was performed in Qiagen's Tissue Lyser TL at 50 Hertz for 5 min. h*ALU* TaqMan RT‐PCR was performed on a Stratagene Mx3005P real‐time instrument using the forward primer 5′‐GGTGAAACCCCGTCTCTACT‐3′, reverse primer 5′‐GGTTCAAGCGATTCTCCTGC‐3′) and probe 5′‐[6FAMCGCCCGGCTAATTTTTGTAT[BHQ1]‐3^40^’ in a total volume of 20 μl containing 10 μl TaqMan Universal Master Mix II, no UNG (Thermo Fisher; Dreiech, Germany; #10380155), 0.3 μM forward and reverse primers, 0.25 μM hydrolysis probe and the corresponding amounts of genomic DNA. The following PCR conditions were used: 1 cycle of 95°C for 10 min, followed by 50 cycles of 95°C for 15 s 56°C for 30 s and 72°C for 30 s. Control RT‐PCR of murine SINEs *B2* was performed using ABsolute QPCR SYBR Green Mix (Life/ThermoFisher; Dreiech, Germany; #AB‐1158B) and the primers 5′‐CAATTCCCAGCAACCACATG‐3′ (forward) and 5′‐ACACACCAGAAGAGGGCATCA‐3′ (reverse).^41^ PCR conditions were one cycle at 95°C for 10 min, followed by 40 cycles at 95°C for 15 s, 56°C for 45 s and 72°C for 30 s.

### 2.22 Mouse model and PET/CT imaging

Tumour cell spheroids generated as for the ex vivo model described above were injected intraperitoneally (i.p.) into immune‐deficient BALB/c‐nude mice (CanN.Cg‐Foxn1nu/Crl; Charles River Laboratories; Sulzfeld, Germany) and the development of metastases was observed at day 28 post injection. One hour before application of 18F‐FDG, mice were placed on a 37°C warm surface to minimise consumption of 18F‐FDG by brown adipose tissue. To avoid interference of 18F‐FDG uptake by insulin and to reduce consumption by the myocardium, animals were deprived of food for a period of 4 h before application. 18F‐FDG was administered i.v. at a dose of 10 MBq in 100 μl of 0.9% NaCl solution into the tail vein 1 h prior to PET/CT imaging under isoflurane anaesthesia for maximally 60 min using a preclinical scanner from Mediso (NanoScan; Mediso Medical Imaging Systems; Budapest, Hungary). Respiration and temperature were observed during the complete acquisition period. Prior to PET a CT scan was performed for each measurement (5 min) for attenuation correction. PET data were acquired with an energy window of 400–600 keV and a coincidence time window of 5 ns. The data were reconstructed using Teratomo 3D from Mediso with a binning of 1:3. A matrix size of 212 × 212 × 239 (voxel: 0.4 mm) was used. CT data (50 kVp, 630 μA, 480 views over 360°) were acquired in one rotation. PET/CT images were reconstructed using a Tera‐TomoTM 3D algorithm (Nucline 3.01.020.000; Mediso Medical Imaging Systems, Budapest, Hungary) with four iterations and six subsets. Co‐registration was performed with the software InterView Fusion (Version 3.01.016.0000).

### 2.23 Statistical analysis of experimental data and functional annotations

Comparative data were statistically analysed by paired or unpaired Student's *t*‐test (two‐sided, unequal variance), as indicated in the figure legends. Statistical significances are indicated as follows: *p < .05; **p < .01; ***p < .001; ****p < .0001. Box plots with medians, upper and lower quartiles, range and outliers were constructed using the Seaborn boxplot function with Python. Functional annotations by gene ontology enrichment analysis or PANTHER classification^42^ were performed using the online tool at http://geneontology.org. Reactome analysis was carried out at https://reactome.org.

## 3 RESULTS

### 3.1 A specific role for BCAM in OC

Analysis of the published TCGA transcriptome dataset^43^ revealed that OC exhibits the highest expression of *BCAM* mRNA of all human malignancies (Figure S1A). Expression of *BCAM* mRNA is associated with both a poor OS (Figure S1B) and RFS (Figure S1C). Intriguingly, *BCAM* expression is significantly associated with a short OS of only two other cancers among the 39 entities in the PRECOG database (Figure S1B; red bars), pointing to a specific role for BCAM in OC. Gene ontology enrichment analysis of genes associated with a poor OS of different human adenocarcinoma revealed ECM organisation and integrin signalling as the most significant term specifically for OC (Table 1), as did Reactome analysis (*p* = 1.9 × 10e17). PANTHER classification^42^ of the same gene set identified integrin signalling as the most significant pathway for OC (*p* = 4.9 × 10e−9). Taken together with the published observations on BCAM–LAMA5–integrin interactions,^5^ these data point to a potential connection between a poor clinical outcome of OC and a role for BCAM in ECM‐mediated signalling.

**TABLE 1:** Functional annotation of genes associated with a poor overall survival (OS) of human adenocarcinomas. Genes with a *z*‐score > 2 (i.e., *p* < .05 and hazard ratio > 1) were retrieved from the PRECOD dataset and analysed for enrichment of GO biological processes. The table lists the top term for each instance, including the number of enriched genes, the fold enrichment and the FDR. Only terms with a fold enrichment ≥ 2.5 and an FDR < 0.01 were included. The data for OC are highlighted in red.

Entity	GO biological process	*n*	Fold	FDR
Bladder	Mitotic cell cycle	128	3.52	1.2E−26
Breast	Mitotic cell cycle	231	2.50	7.5E−22
Colon	Vasculature development	67	2.64	1.1E−08
Liver	Regulation of intrinsic apoptotic signalling pathway	23	3.59	5.5E−04
Lung	Mitotic cell cycle	206	2.64	2.8E−26
Ovarian	ECM organisation	77	2.80	2.2E−10
Pancreatic	Cellular response to DNA damage stimulus	76	2.59	8.1E−10
Prostate	Mitotic cell cycle	67	4.40	4.2E−14

### 3.2 Characterisation of soluble BCAM in OC ascites

**FIGURE 1:**
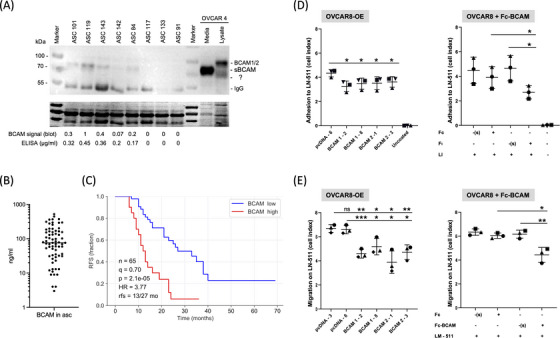
Analysis of BCAM in OC ascites and comparative functional analysis of membrane‐bound and soluble BCAM. (A) Immunoblot of BCAM in eight different cell‐free ascites samples. For comparison, conditioned medium (containing sBCAM) and lysate from OVCAR4 cell was included (right‐most lanes). Quantitation of relative signal intensities as well as BCAM levels in ascites samples measured by ELISA are shown at the bottom. The band labelled with ‘?’ denotes an unspecific background band. The bottom panel shows the membrane after staining with the Pierce Reversible Total Protein Stain Kit as loading control. (B) Concentration of BCAM protein in the ascites from *n* = 70 high‐grade serous OC patients determined by ELISA. (C) Kaplan–Meier plot analysing the relapse‐free survival (RFS) of *n* = 65 evaluable patients analysed in panel B. Groups were split at the *q* = 0.7 quantile (best‐fit); *p*: logrank *p* value; HR: median hazard ratio; rfs: months to 50% RFS for patients with high/low BCAM levels. (D) Effect of BCAM on OC cell adhesion to LN‐511 on non‐adhesive microplates coated with LN‐511. Cell adhesion was quantified by RTCA. Left: BCAM‐overexpressing OVCAR8 cells (OVCAR8‐OE). Clones stably transfected with BCAM1 or BCAM2 (Figure S4) were compared with cells transduced with the empty expression vector (pcDNA‐6). Right: Adhesion of OVCAR8 cells was analysed in the presence of Fc‐BCAM or negative control (Fc) at equimolar concentration (1 μg/ml of Fc‐BCAM; 0.33 μg/ml of Fc). (s): solvent for Fc or Fc‐BCAM. (E) Effect of BCAM on two‐dimensional OC cell migration under the same conditions as in panel D, except that a further control clone (pcDNA3) was included. Transwell‐chamber microplates were coated with LN‐511 and cell migration was quantified by RTCA. The data in D and E are based on *n* = 3 biological replicates. *p < .05; **p < .01; ***p < .001; ns, not significant by unpaired *t* test.

The source and potential functions of soluble BCAM in cancer are unknown. To elucidate the nature of ascites‐associated BCAM in OC patients we performed immunoblotting experiments, which revealed only traces of, if any, BCAM1/2 with an apparent molecular mass of 95 kDa, while a shorter BCAM form of approximately 80 kDa was abundant (Figure 1A). Consistent with these findings, a short BCAM form of very similar length was observed in conditioned medium from OVCAR4 cells, while BCAM1/2 was detected exclusively in whole cell extracts (Figure 1A, rightmost lanes). The signal intensities for the shorter BCAM form in ascites correlated well with the BCAM levels measured by ELISA (Figure 1A, bottom). We will henceforth refer to this shorter soluble BCAM form in ascites and conditioned medium as sBCAM. ELISA measurements of *n* = 70 ascites samples of a high‐grade serous OC patient cohort^25^ revealed a wide concentration range for sBCAM of 0–520 ng/ml with a median level of 76 ng/ml (Figure 1B), and ascites levels of sBCAM were significantly associated with a short RFS (Figure 1C), consistent with a previous aptamer‐based analysis.^25^ Correlation analyses showed a link between the levels of BCAM protein in tumour cells from ascites^29^ (ascTU) and the level of sBCAM in ascites (Spearman rho = 0.78; *p* = .014), suggesting that the expression level of BCAM, besides shedding proteases, is one of the factors determining the concentration of sBCAM. In tumour cells from ascites, *BCAM1* RNA was the predominant form in most samples, with a highly variable *BCAM1/BCAM2* ratio ranging from 0.8 to 50 (Figure S2).

### 3.3 Comparative functional analysis of membrane‐bound and soluble BCAM

Previous studies with 3T3 fibroblasts,^7^ HT1080 osteosarcoma cells,^5^ gastric cancer cells,^8^ rat hepatoma cells^18^ and MDCK cells^19^ showed that BCAM can modulate cell adhesion and migration on LN‐511 matrices (see *Background* for details). To compare the biological properties of the BCAM forms we therefore analysed their effect on the adhesion and migration of OC cells. LN‐511 deserves particular attention due to its prominence in the OC TME, as suggested by the high RNA expression of its subunits in tumour and/or tumour‐associated cells^30^ (Figure S3A), which is mirrored by OVCAR cell lines (Figure S3B).

As a source of soluble BCAM protein for functional assays, we used recombinant Fc‐BCAM expressed in mammalian cells. Fc‐BCAM is composed of a fragment lacking the transmembrane and the intracellular domains fused to an Fc fragment. Importantly, Fc‐BCAM retains an intact LAMA5 binding site.^15^ Fc‐BCAM was used at a concentration of 1 μg/ml, which approximately corresponds to the highest molar concentration of sBCAM in ascites (Figure 1B). The impact of different BCAM forms on matrix adhesion was quantified by xCELLigence‐based RTCA.^44^ Overexpression of BCAM1 or BCAM2 (Figure S4A; cell surface localisation verified in Figure S4B) or exposure to Fc‐BCAM resulted in an inhibition of adhesion to LN‐511 (Figure 1D). As reduced adhesion of BCAM‐overexpressing cells was observed as soon as attachment was detectable (∼15 min after plating; Figure S7), it is likely that non‐cleaved, membrane‐bound BCAM contributes to the inhibitory effect. Consistent with gain‐of‐function experiments in Figure 1D, *BCAM* gene disruption (Figure S5A) or inhibition of BCAM expression by siRNA‐mediated interference (Figure S5B) enhanced adhesion (Figures S6C and D).

Analysis of adhesion to LN‐511 of 3 additional vector control clones and wildtype OVCAR8 cells (not shown) yielded results identical to the control clone used in Figure 1D (pcDNA‐6), thus strongly reducing the probability of experimental artefacts due to clonal selection. Moreover, no significant effects of BCAM were observed on the adhesion of OC cells to COL1 (Figure S6E–G), which may be relevant with respect to its known role in OC cell invasion.^45^, ^46^, ^47^, ^48^, ^49^, ^50^

Similar to its effect on adhesion, overexpression or the addition of Fc‐BCAM (Figure 1E) inhibited migration, while *BCAM* disruption resulted in enhanced migration (Figure S8C). Furthermore, real‐time microscopic analyses of undirected motility of BCAM‐overexpressing and Fc‐BCAM‐treated OVCAR8 cells revealed a clear inhibitory effect on both cases (Figures S8D and E).

Our findings are consistent with the known interaction of BCAM with LAMA5 (the LN‐511 alpha subunit), which has been proposed to interfere with LAMA5 binding to integrins.^5^ Importantly, BCAM1 and BCAM2, the latter lacking most of the intracellular domain, showed nearly identical effects, suggesting that BCAM does not act as a signalling receptor to mediate inhibition of LN‐511‐dependent adhesion. This conclusion is supported by the remarkably similar effect of Fc‐BCAM and the membrane‐associated BCAM proteins.

### 3.4 Metalloproteinases produced by OC cells

**FIGURE 2:**
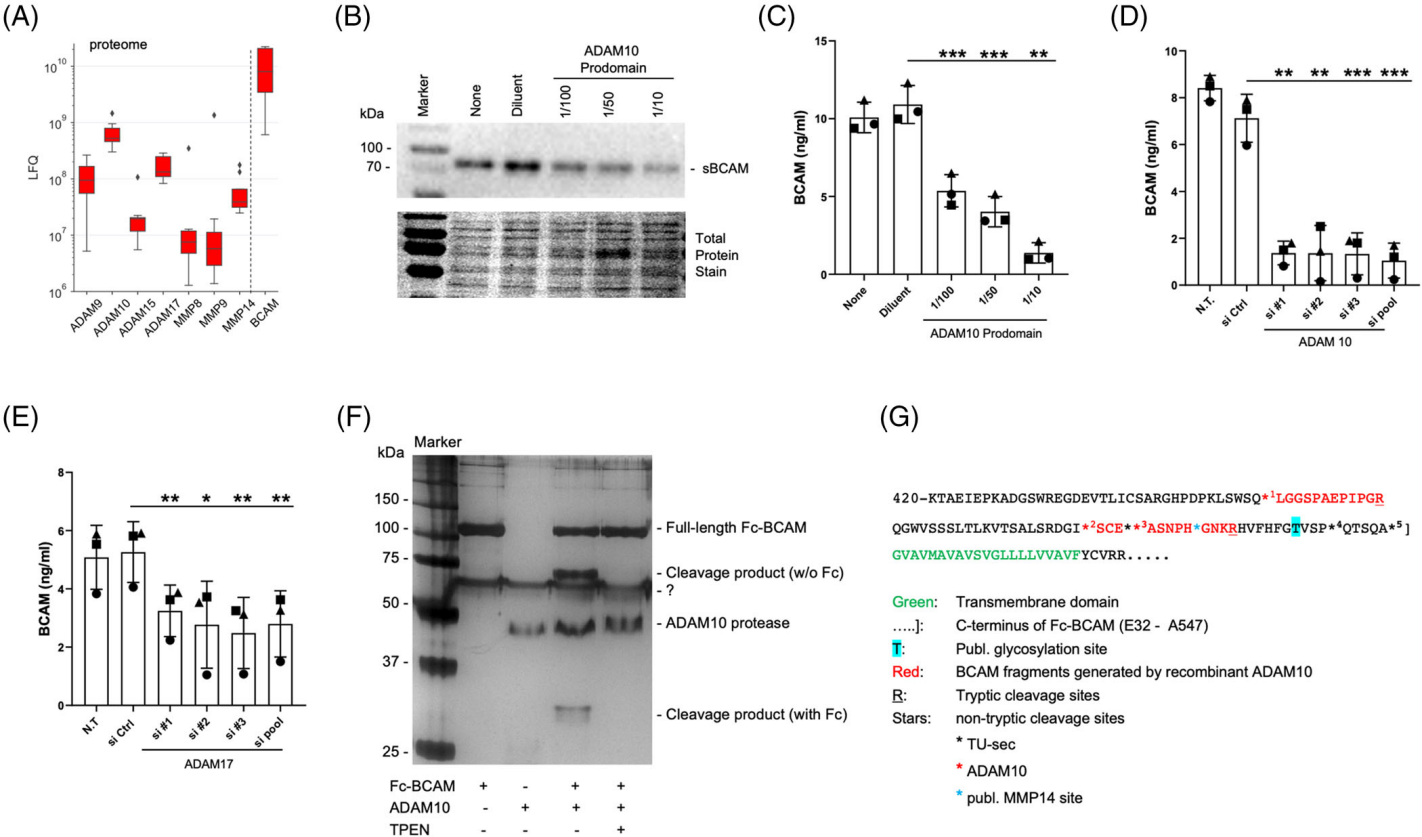
Role of ADAM10 in the generation of sBCAM and identification of cleavage sites. (A) Distribution of BCAM and metalloproteinase expression in tumour cells from *n* = 9 OC patients, based on a previously published dataset obtained by MS‐based proteomic analysis.^5^ Signal intensities reflect LFQ values. Boxplots show medians (line), upper and lower quartiles (box), ranges (whiskers) and outliers (diamonds). (B) Immunoblot of medium from OVCAR4 cells cultured in the presence of different concentrations of the ADAM10 prodomain (selective ADAM10 inhibitor)^51^ for 24 h. The panel below the immunoblot show the respective membranes stained with the Pierce Reversible Total Protein Stain Kit. (C) ELISA‐based quantification of sBCAM secretion by OVCAR4 cells treated as in panel B. (D) Analysis of BCAM release by OVCAR4 cells treated with three different siRNAs targeting ADAM10 (s1#1, si#2, si#3), a pool of all three siRNAs (si pool) or negative control siRNA (si Ctrl). The leftmost bar represents non‐transfected cells (NT). Cell culture media were analysed by ELISA as in panel C. (E) Analysis of OVCAR4 cells as in panel D but treated with ADAM17‐targeting siRNAs. *p < .05; **p < .01; ***p < .001 by unpaired *t*‐test. (F) Silver‐stained PAGE gel of recombinant Fc‐BCAM after digestion with recombinant ADAM10 and in the absence or presence of the zinc chelator TPEN. ‘?’ denotes an unspecific background band. (G) Schematic representation of the C‐terminal amino acid sequence of BCAM, including the ADAM10 cleavage sites in recombinant BCAM identified in panel F (Table S3), the cleavage sites found in the secretome of tumour cells from OC ascites (TU‐sec; Table S4) and the previously published MMP14 cleavage site.^26^

The data in Figure 1A suggest that sBCAM may be generated by shedding through proteolytic cleavage of the membrane‐bound forms. To identify candidate BCAM sheddases, we analysed our published proteomics datasets^29^ of tumour cells from OC ascites for expression of metalloproteinases of the ADAM and MMP families. As shown in Figure 2A, four ADAMs (ADAM9, 10, 15, 17) and three MMPs (MMP8, 9, 14) were identified in whole cell proteomes. The highest expression levels were observed for ADAM10 and ADAM17, raising the possibility that they might represent BCAM‐cleaving proteases. In agreement with this result, ADAM9, 10 and 17 were also found in the secretome of these cell types, with the highest expression levels observed for ADAM10 (Figure S9A).

To gauge the suitability of OC cell lines as experimental models we analysed the expression of *ADAM* genes in OVCAR3, OVCAR4, OVCAR5 and OVCAR8. As shown in Figure S9B, all four cell lines expressed *ADAM9, ADAM10, ADAM15* and *ADAM17* at levels of ∼40–370 TPM, while transcripts from all other ADAM genes were much lower or undetectable. This pattern is consistent with the proteome data for primary tumour cells described above (Figure 2A), particularly for OVCAR4, OVCAR5 and OVCAR8 (low ADAM15 expression). These three cell lines also expressed the active form of ADAM10 (Figure S10A). However, OVCAR5 cells exhibited barely detectable expression of BCAM protein (Figure S10B), which is inconsistent with the in vivo findings (Figure 2A), and were therefore excluded from further analyses.

### 3.5 Identification of ADAM10 as the major BCAM sheddase of OC cells

Production of sBCAM by OVCAR‐4 cells was strongly inhibited by the broad‐spectrum metalloproteinase inhibitor marimastat in a concentration‐dependent fashion, as shown by immunoblotting and ELISA (Figures S11A and B) of conditioned medium (85% inhibition at 1 μM). A similar pattern was observed with a recombinant ADAM10 prodomain, a specific inhibitor of ADAM10^51^ (Figures 2B and C; 88% inhibition at the lowest dilution), pointing to a significant role for ADAM10 in shedding BCAM from OC cells. This was confirmed by siRNA‐mediated interference with ADAM10 expression (Figures 2D and S10C), showing 85% inhibition similar to the ADAM10 prodomain. Interference with ADAM17 expression also significantly inhibited the release of sBCAM but to a considerably lower extent (35% inhibition; Figures 2E and S10D). These data strongly suggest that both ADAM10 and ADAM17 contribute to BCAM shedding, with ADAM10 as the major contributor.

### 3.6 ADAM10 cleavage sites in BCAM protein

Cleavage of BCAM by ADAM10 was reproduced using recombinant proteins. As shown in Figure 2F and Tables S2 and S3, incubation of recombinant Fc‐BCAM (see Figure 2G for details) with recombinant ADAM10 yielded two fragments, which disappeared in the presence of the zinc chelator and ADAM10 inhibitor TPEN, indicating the specificity of the observed cleavage. Mass spectrometry (MS) identified three cleavage sites near the transmembrane domain (red stars in Figure 2G; sites 1, 2 and 3). MS analysis also identified three cleavage sites in BCAM in the conditioned medium from primary OC cells (black stars in Figure 2G; sites 3, 4 and 5), one of which (site 3) coincides with a cleavage site found with recombinant ADAM10. A published MMP14 cleavage site^26^ is also indicated (blue star in Figure 2G).

To gain further insight into the role of ADAM10 in BCAM cleavage we sought to align sites 1–5 with known ADAM10‐targeted motifs. As ADAM10 activity is highly promiscuous, a clear consensus sequence of its targeted cleavage sites has not been defined. We therefore made use of two published unbiased screening approaches identifying preferred amino acids surrounding ADAM10 cleavage sites.^52^, ^53^ We combined these datasets to compile the table in Figure S12A, which also distinguishes between strongly and weakly enriched amino acids. Figure S12B shows alignments of cleavage sites 1–5 in BCAM with these published data. Sites 3–5 show considerable matches, consistent with ADAM10‐mediated cleavage. It is possible that sites 4 and 5, which are cleaved in vivo, are missing in the in vitro experiment, as they are located close to the C‐terminus of the BCAM fragment and the Fc fusion (see Figure 2G). By contrast, sites 1 and 2 identified in cleaved Fc‐BCAM barely matching the published amino acid preferences (Figure S12B) were not found in vivo and therefore likely play a minor role, if any, in the shedding of BCAM by ADAM10. This suggests that the sites proximal to the transmembrane domain (site 3 and possibly 4 and 5) are the major sites cleaved by ADAM10 to produce sBCAM from OC cells.

Our findings together with published data indicate that BCAM is cleaved by at least three proteases (ADAM10, ADAM17, MMP14) at multiple adjacent sites, suggesting that sBCAM in ascites (Figure 1A) represents a mixture of fragments with different C‐termini. Although the exact lengths of these proteins with a relative molecular mass of 70 kDa are not known, it is highly likely that they retain the LAMA5‐binding site, which is located far away from the cleavage sites and functionally relevant in the context of our study.

### 3.7 Immunohistochemical visualisation of BCAM and matrix proteins in OC metastasis and spheroids from OC ascites

**FIGURE 3:**
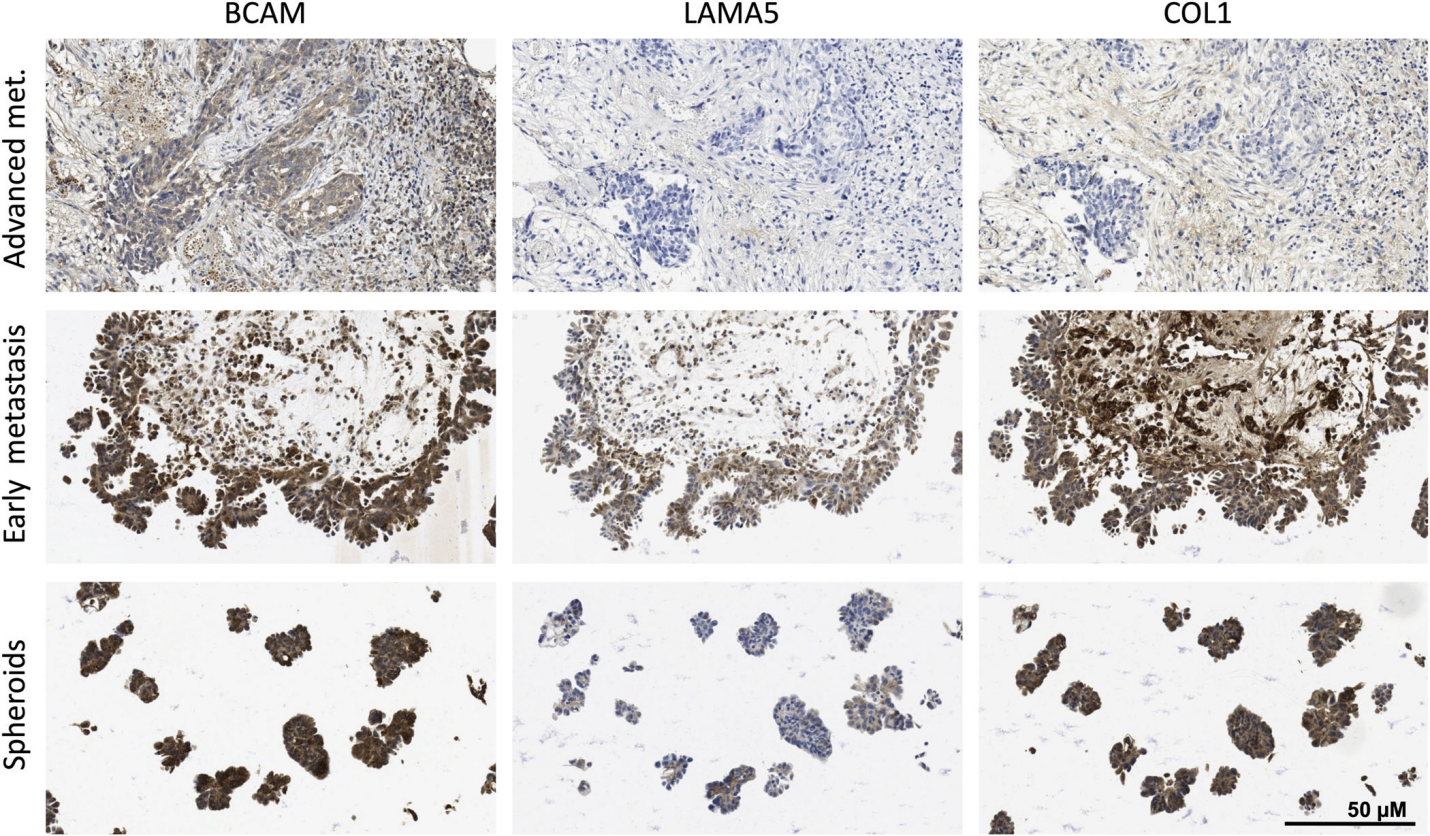
Immunohistochemical analysis of BCAM, LAMA5 and COL1 in matched samples of OC metastases and spheroids from ascites. Paraffin sections from metastases at different stages (early metastases: tumour cells still near the surface; advanced metastases: deeply invading larger tumour masses) and spheroids from ascites were stained by immunohistochemistry as described in *Materials and Methods*. A quantification of the images is shown in Table 2 (patient OC114). Further examples are depicted in Figure S13 and quantified in Table 2. Scale bar: 50 μm

To be able to interpret the results obtained in the present study in the context of OC metastasis we analysed BCAM, the LN‐511 α‐subunit LAMA5 and COL1 in metastases and ascites spheroids from eight patients (clinical data in Table S2) by immunohistochemistry. The results of this analysis are summarised in Table 2 and examples are presented in Figures 3 and S13 and can be summarised as follows:

1. BCAM is expressed in tumour and stroma compartments of metastasis as well as in spheroids, albeit at varying levels;
2. LAMA5 expression is observed in all spheroids in contrast to metastases (two out of eight positives in tumour compartment; stroma compartment negative in all samples except for a few small areas with positive cells in two cases; see Table 2);
3. Tumour cells in early metastases (small and near the surface) from patient OC114 are LAMA5‐posivive, and in this respect resemble spheroids more closely than advanced metastases, supporting the notion that LAMA5 protein or accessibility may be regulated during metastatic growth in some cases;
4. COL1 is clearly expressed in the stroma compartments of all tumour samples and all spheroids; expression is weaker in the tumour cell compartments.

**TABLE 2:** Summary of immunohistochemical analysis of BCAM, LAMA5, COL1 and COL4 in matched samples of OC metastases (Met) and spheroids from ascites (Asc). Staining intensities were classified as follows: Negative (0, black), weak (1, blue), moderate (2, brown) and strong (3, red). Metastatic sites analysed are listed in Table S2. Examples are shown in Figures 3 and S13

Patient ID	BCAM			Laminin α5 (LAMA5)			Collagen I (COL1)			Collagen IV (COL4)		
	Met tumour	Met stroma	Asc spheroid	Met tumour	Met stroma	Asc spheroid	Met tumour	Met stroma	Asc spheroid	Met tumour	Met stroma	Asc spheroid
OC26	**3**	**2**	**3**	**0**	**0**	**3**	**1**	**2**	**3**	**0**	**1**	**0**
OC27	**3**	**2**	**3**	**0**	**0**	**1**	**1**	**2**	**2**	**0**	**1**	**0**
OC54	**1**	**1**	**2**	**0**	**0**	**2**	**1**	**2**	**2**	**0**	**1**	**0**
OC66	**1**	**1**	**3**	**0**	**0**	**2**	**0**	**1**	**2**	**0**	**1**	**0**
OC67	**2**	**2**	**3**	**0**	**0**	**2**	**1**	**1**	**2**	**0**	**1**	**0**
OC84	**3**	**2**	**3**	**3**	**0**	**2**	**1**	**3**	**2**	**0**	**1**	**1**
OC114*	**1**	**1**	**3**	**0**	**0**	**2**	**0**	**1**	**3**	**0**	**1**	**0**
OC122	**2**	**2**	**3**	**2**	**0**	**1**	**1**	**2**	**2**	**0**	**1**	**0**

^*^Small metastases from patient OC114 were positive of LAMA5 (score 2; see Figure 3).

COL1 and LAMA5 were found majorly as deposits around cells, particularly in spheroids, while BCAM antibodies stained both the plasma membrane and cytoplasm, albeit with inter‐sample variability (Figure S13). These results agree with the proposed essential role for COL1 in OC metastasis.^45^, ^46^, ^47^, ^48^, ^49^, ^50^ The data also reveal a potential role for LAMA5 selectively in spheroids with implications for BCAM. For comparison, we also analysed the expression of the basal membrane protein collagen IV (COL4), which was not detectable in the tumour compartment of all metastases and spheroids (examples in Figure S13 and Table 2).

### 3.8 Impact of BCAM on OC spheroid formation

**FIGURE 4:**
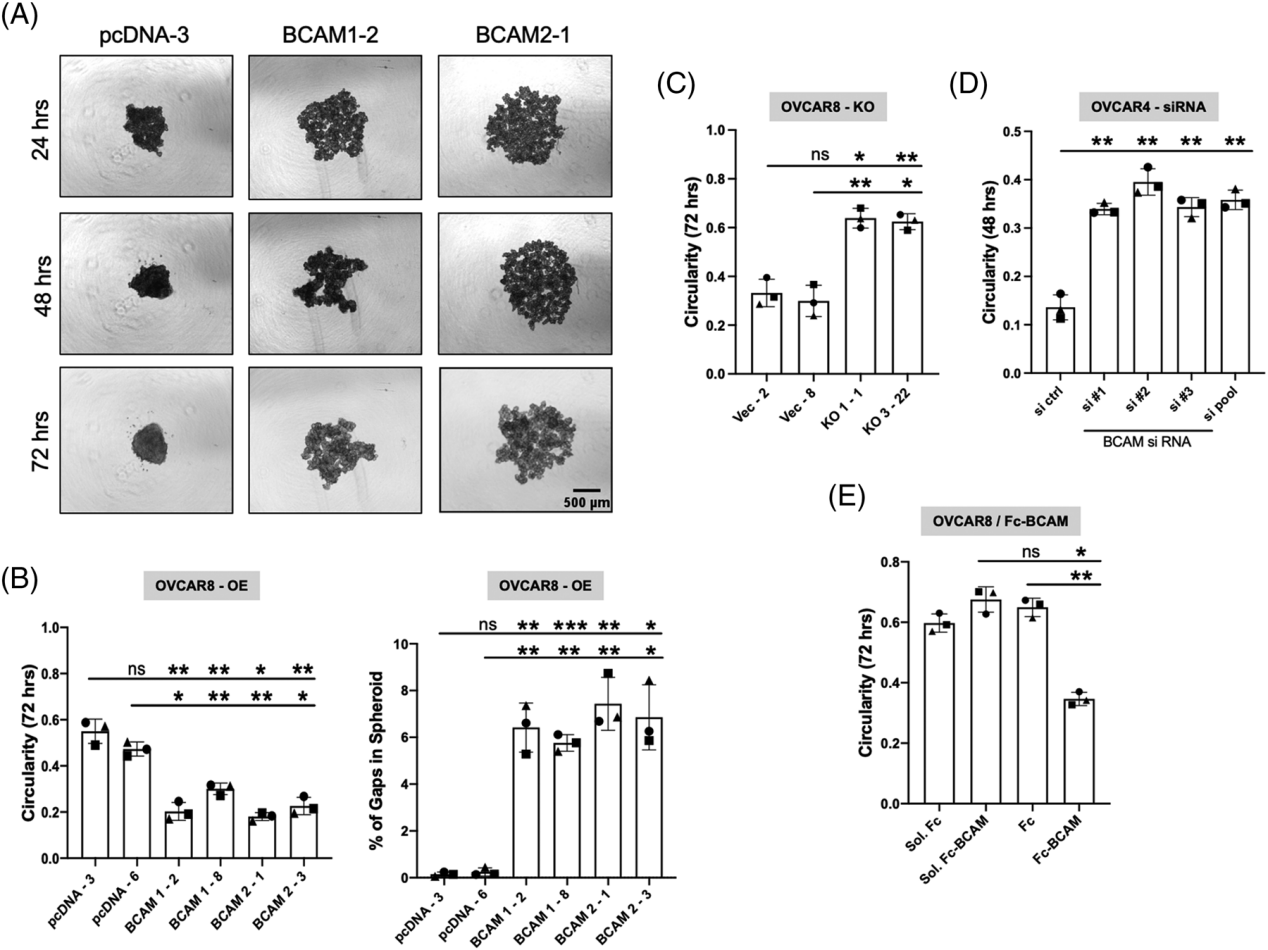
Impact of BCAM on OC cell spheroid formation. (A) Morphology of spheroids derived from BCAM‐overexpressing OVCAR8 clones (BCAM1‐2, BCAM2‐1) compared with cells transduced with the empty pcDNA‐3 vector (representative examples). Scale bar: 500 μm. (B) Circularity of spheroids and percentage of gaps in spheroids as in panel A. The plot shows quantifications for two different clones in each case. (C) OVCAR8 cells with disrupted BCAM (OVCAR8‐KO) compared with cells transduced with the empty vector (clones Vec‐2, Vec‐8). The plot shows circularities for two different clones in each case. (D) OVCAR4 cells transfected with control‐siRNA (si ctrl), three different BCAM‐siRNAs (#1, #2, #3) or pooled siRNAs (pool). (E) Spheroids from OVCAR8 cells formed in the presence of Fc‐BCAM or Fc control at equimolar concentration (1 μg/ml of Fc‐BCAM; 0.33 μg/ml of Fc). Sol: solvent for Fc or Fc‐BCAM. Each plot is based on *n* = 3 biological replicates. *p < .05; **p < .01; ***p < .001; ns: not significant by unpaired *t*‐test

The inhibitory effects of BCAM on known functions of tumour cells, that is, adherence and migration of single cells (Figures 1D, 1E, S6–S8), cannot explain its association with a poor clinical outcome (Figures 1C and S1). In view of the known pivotal role of spheroids rather than single tumour cells in transcoelomic spreading and the observed deposition of LAMA5‐containing laminin in spheroids (Figure 3), we investigated a potential function of BCAM as a laminin‐interacting protein in spheroid formation and dynamics. Figure 4A depicts the morphology of OVCAR8 spheroids derived from BCAM1‐ and BCAM2‐overexpressing OVCAR8 cells (OVCAR8‐OE) compared with control cells. It is obvious that expression of both BCAM isoforms decreased the compactness of spheroids. This conclusion was confirmed by image analysis of 4 different clones, revealing a significantly decreased circularity (“roundness”) and clearly increased gap formation in spheroids (Figure 4B). In agreement with these observations, genetic disruption (KO) of BCAM in OVCAR8 cells (Figure 4C; OVCAR8‐KO) and BCAM siRNA treatment of OVCAR4 cells (Figure 4D) had the opposite effect. To confirm the role of BCAM in spheroid formation, we tested a third cell line, OVCAR3, which expresses BCAM at a similar level to OVCAR8 (Figure S10B). Consistent with the OVCAR4 data, siRNA‐mediated inhibition of BCAM expression in OVCAR3 cells resulted in a strongly increased compaction of spheroids (Figure S14). Finally, exposure of OVCAR8 cells to Fc‐BCAM emulated the effect of BCAM overexpression, leading to a significant decrease of spheroid compactness (Figure 4E), analogous to our observations in other functional assays described above.

### 3.9 Role of LN‐511 and integrin β1 in BCAM‐regulated spheroid compaction

**FIGURE 5:**
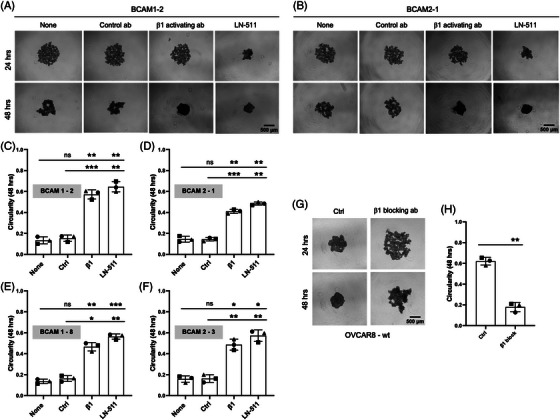
Role of LN‐511 and integrin β1 in BCAM‐regulated spheroid compaction. (A, B) Morphology of spheroids derived from BCAM‐overexpressing OVCAR8 clones (BCAM1 in panel A; BCAM‐2 in panel B) cultured in the presence of an integrin‐β1 activating antibody, a control antibody or exogenous LN‐511 (representative examples). None: untreated cells. Scale bar: 500 μm. (C, D) Quantification of circularity of spheroids in panels A and B (*n* = 3 biological replicates each). (E, F) Circularity of spheroids from two additional BCAM‐overexpressing clones (*n* = 3 replicates). (G) Morphology of spheroids derived from OVCAR8 cells in the presence of an integrin‐β1 blocking antibody or a control antibody (Ctrl). Scale bar: 500 μm. (H) Quantification of circularity of spheroids in panels G. Data are shown for *n* = 3 biological replicates in panels C–F and H. *p < .05; **p < .01; ***p < .001; ns: not significant by unpaired *t*‐test

Considering the results described above we asked whether BCAM might exert its effect on spheroid compaction by interfering with the LAMA5–integrin β1 interaction in spheroids. Several lines of experimental evidence support this notion. As shown in Figures 5A–F, an integrin‐β1‐activating antibody significantly alleviated the inhibitory effect of ectopic BCAM1 or BCAM2 expression in four independent clones in contrast to a control antibody (∼3‐fold increase in circularity) after a 48‐h culture period. Likewise, the addition of soluble LN‐511 also significantly reduced the BCAM‐mediated effect. Furthermore, an integrin‐β1‐blocking antibody inhibited the compaction of wild‐type OVCAR8 spheroids (Figures 5G and H) similar to BCAM overexpression (Figures 4A and B), indicating that integrin accessibility is essential for spheroid formation. These observations strongly suggest that BCAM reduces the compaction of spheroids by competing with integrin β1 for interaction with LAMA5.

### 3.10 Impact of BCAM on spheroid dispersion and mesothelial clearance

**FIGURE 6:**
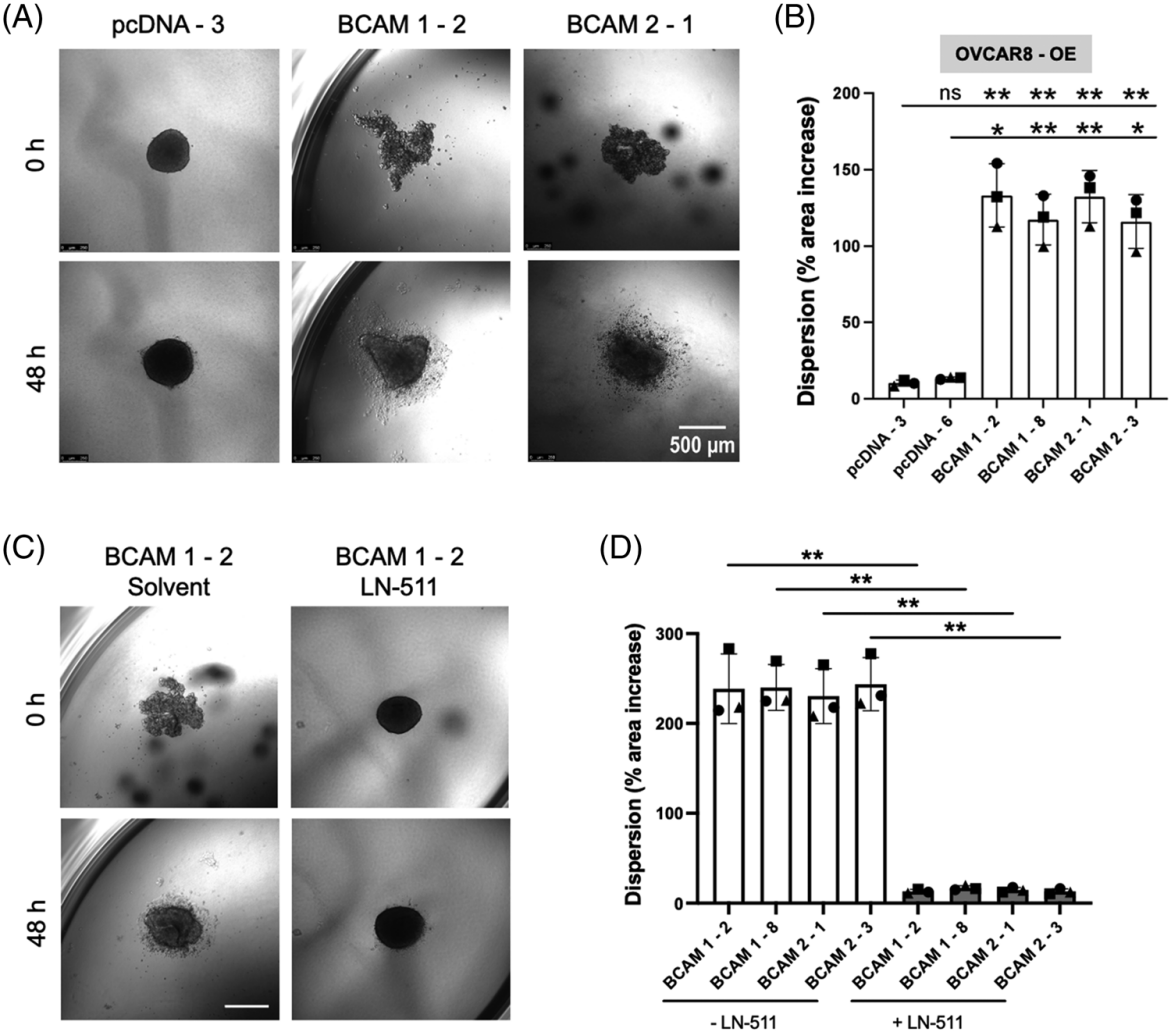
Effect of BCAM on spheroid dispersion. (A) Spheroids of BCAM‐overexpressing OVCAR8 clones (BCAM1‐2, BCAM2‐1) and cells transduced with the empty vector (pcDNA‐3) were embedded in a 3D collagen matrix for 48 h. The photomicrographs show a clear dispersion only for the BCAM‐overexpressing cells. Scale bar: 500 μm. (B) Quantification of dispersion of spheroids in panel A plus one additional clone for each condition (*n* = 3 biological replicates each). *p < .05; **p < .01; ***p < .001; ns: not significant by unpaired *t*‐test. (C) Spheroids were generated from BCAM‐overexpressing BCAM1‐2 cells in the presence or absence of exogenous LN‐511 (10 μg/ml) as in panel A. Pictures were taken at times 0 and 48 h after embedding. (D) Quantification of dispersion of spheroids generated from four different BCAM‐overexpressing and four control clones in the presence or absence of exogenous LN‐511 as in panel C (*n* = 3 biological replicates each). **p < .01 by paired *t*‐test

We next addressed the potential relevance of BCAM's effect on spheroids in the context of transcoelomic metastasis formation. Invasion of peritoneal organs has been described to be critically dependent on spheroid dispersion and clearance of the mesothelial cell layer at attachment sites.^54^ As shown in Figures 6A and B, both BCAM1 and BCAM2 overexpressing OVCAR8‐OE cells showed clearly enhanced dispersion in a collagen matrix compared with vector control cells using 2 different clones for each condition (>100% enlargement of area relative to the initial spheroid after 48 h versus 10–20%). Moreover, exogenous LN‐511 added to developing spheroids blocked BCAM‐induced spheroid dispersion (Figures 6C and D), suggesting that the role of BCAM in spheroid dispersion is related to its inhibitory impact on LAMA5‐dependent spheroid compaction shown in Figure 5.

**FIGURE 7:**
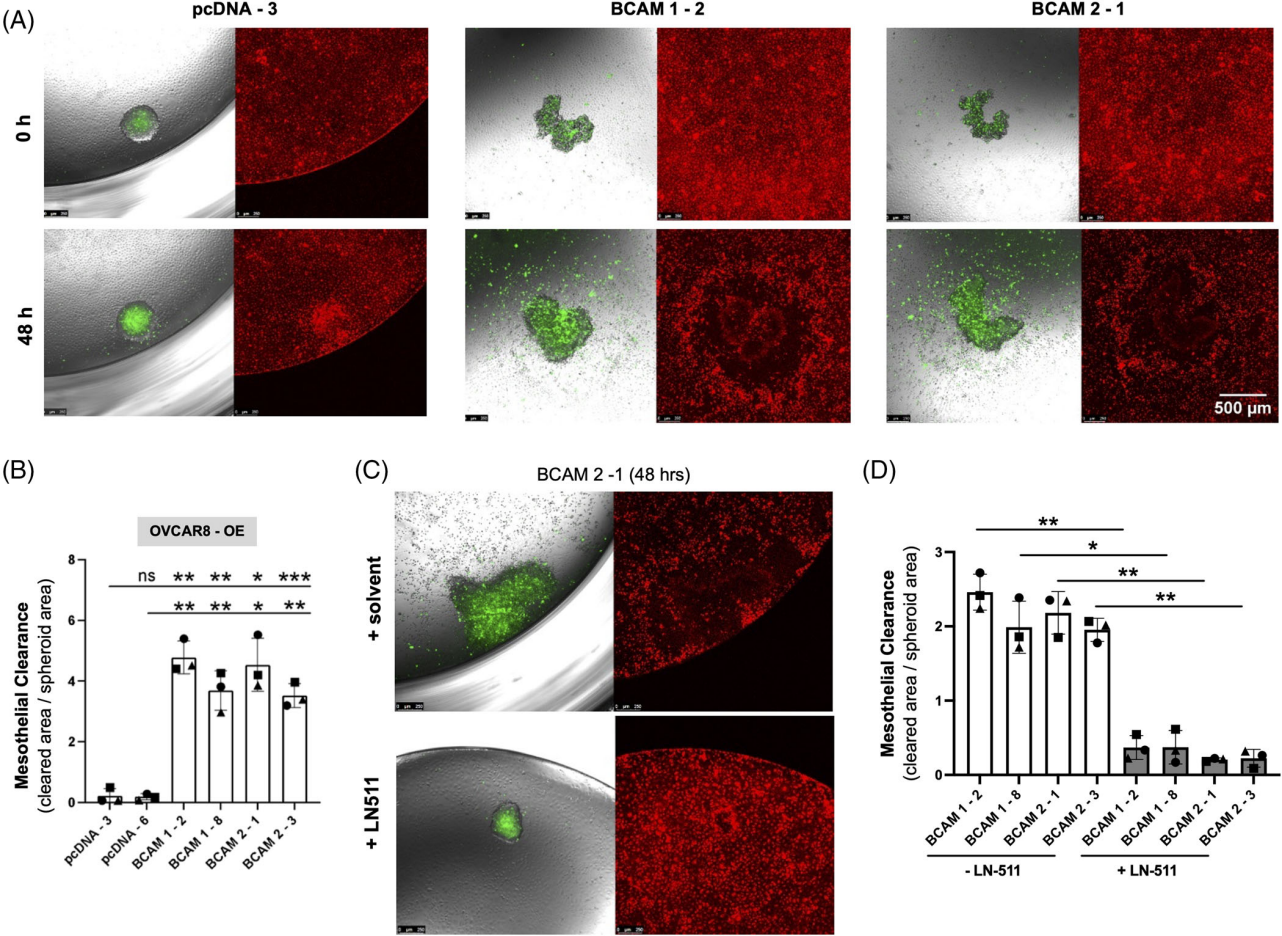
Effect of BCAM on clearance of a mesothelial monolayer. (A) The same clones as in Figure 6 (labelled with Cell Tracker green) were plated on a confluent monolayer of omental mesothelial cells (Cell Tracker orange) and mesothelial cell clearance was observed after 48 h. Scale bar: 500 μm. (B) Quantification of mesothelial cell clearance by clones in panel A plus one additional clone for each condition (*n* = 3 biological replicates each). *p < .05; **p < .01; ***p < .001; ns: not significant by unpaired *t*‐test. (C) Spheroids were generated from BCAM‐overexpressing BCAM2‐1 cells in the presence or absence of exogenous LN‐511 (10 μg/ml) as in panel A and analysed after 48 h. (D) Quantification of mesothelial cell clearance by spheroids generated from four different BCAM‐overexpressing and four control clones in the presence or absence of exogenous LN‐511 as in panel C (*n* = 3 biological replicates each). *p < .05; **p < .01 by paired *t*‐test

Mesothelial cell clearance is a step strongly associated with the adhesion to and invasion of peritoneal organs. We therefore addressed the potential role of BCAM in this process using co‐cultures of spheroids with mesothelial monolayers. As shown in Figure 7A, both BCAM1‐ and BCAM2‐overexpressing OVCAR8‐OE cells (labelled green) efficiently induced gaps in the mesothelial cell monolayer (labelled red) in contrast to control (pcDNA) cells. Very similar results were obtained with two independent clones for each condition (Figure 7B). To exclude potential effects of soluble BCAM shed from BCAM‐OE cells, we also tested the effect of Fc‐BCAM on mesothelial cells, which did not induce any detectable changes (data not shown). Finally, addition of LN‐511 during spheroid formation blocked BCAM‐induced mesothelial clearance (Figures 7C and D), presumably as a consequence of diminished spheroid dispersion (Figure 6).

Taken together, these observations strongly suggest that BCAM‐mediated dispersion of spheroids promotes trans‐mesothelial invasion, probably due to weakening of integrin‐β1‐LAMA5‐mediated intraspheroidal cohesion of tumour cells.

### 3.11 Effect of BCAM on OC cell invasion into the omentum

To validate the in vitro observations described above we used an ex vivo model of mouse omentum adapted from Khan and colleagues.^36^ A prerequisite for obtaining conclusive data from ex vivo models is the integrity of the organ under investigation during the observation period. This applies particularly to mesothelial cells which are prone to activation and premature senescence under stress conditions.^55^ We found that under standard culture conditions the activation marker VCAM1 strongly and steadily increased in the mesothelial cells of mouse omentum cultured ex vivo for 1–5 days (Figure S15A), indicating an aberrant state of the tissue. It has been shown that advanced glycation end‐products (AGEs) induce VCAM1 in mesothelial cells by binding to their receptor RAGE.^38^ AGEs are formed under hyperoxic conditions^56^ such as those in regular cell culture. We therefore tested the effect of delipidised serum instead of regular FCS under hypoxic conditions. As shown in Figure S15B, the aberrant induction of VCAM1 was strongly diminished under these conditions, which were therefore used in all subsequent experiments.

**FIGURE 8:**
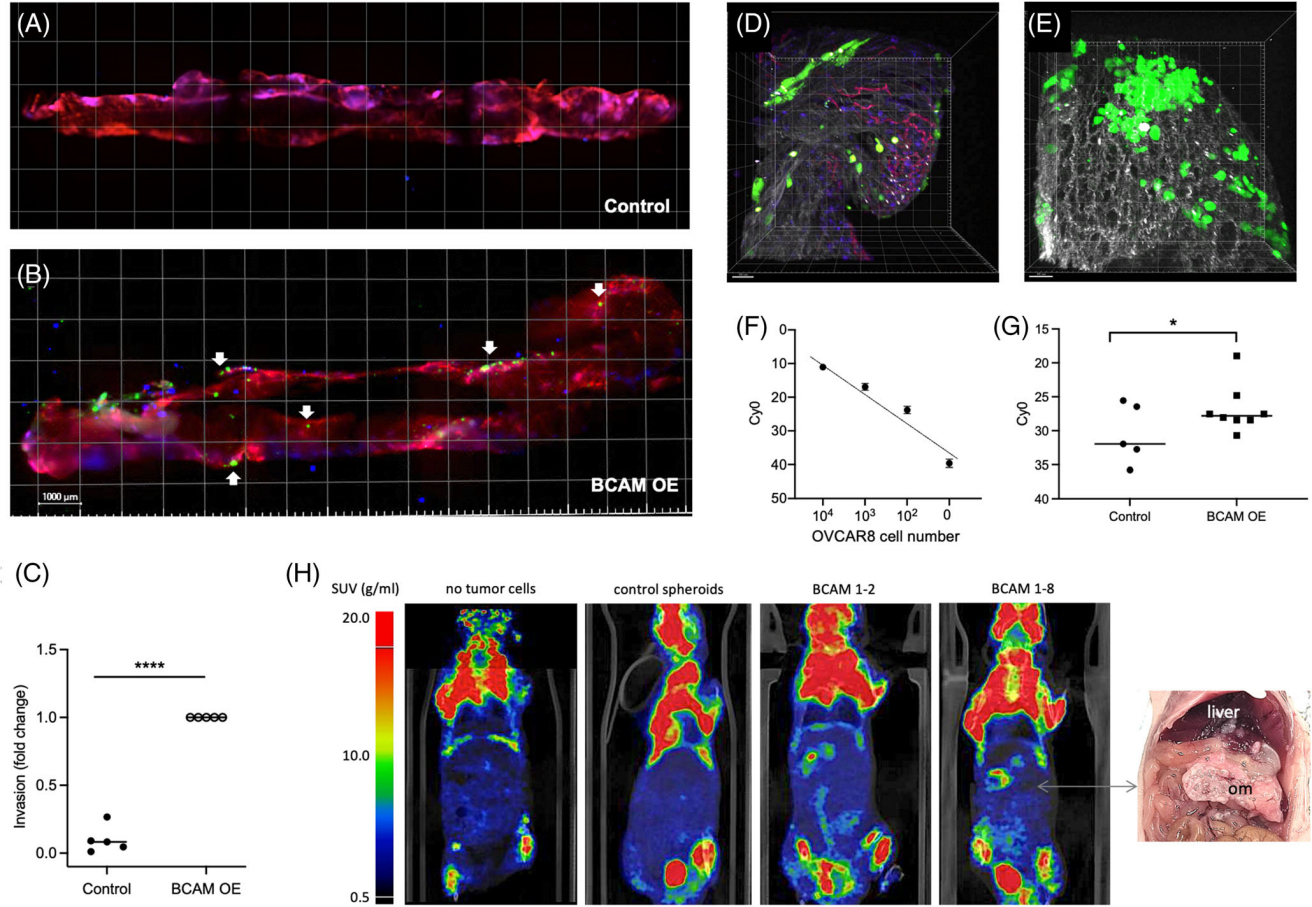
Effect of BCAM on invasion of mouse omentum by tumour cell spheroids. (A, B) Representative light‐sheet microscopic images showing the invasion of explanted mouse omentum ex vivo by spheroids derived from control (A) and BCAM1‐overexpressing (B) OVCAR8 cells pre‐labelled with Cell Tracker Green. Spheroids derived from equal numbers of cells were added to freshly resected omentum and co‐cultured for 48 h. Thereafter, the omentum was stained for immune cells (CD45; blue) and microvessels (CD31; red) and observed by light‐sheet microscopy. Arrows point to areas of tumour cells that are not in the vicinity of milky spots (examples). These areas are characterised by the absence of CD45+ cell clusters (blue), which appear purple if co‐localising with CD31+ endothelial cells. Scale bar: 1000 μm. The sharp blue spots represent staining artefacts. (C) Quantification of the number of invaded cells analysed as in panels A and B for n = 5 biological replicates. ****p < .0001 by t test. (D, E) Multiphoton microscopy of tumour cells from BCAM‐overexpressing OVCAR8 spheroids pre‐labelled with Cell Tracker Green. Collagen fibres are visualised in white by second‐harmonic generation. Panel D shows the area below a milky spot, panel E an area distant from milky spots. Scale bar: 50 μM. (F) Validation of Taqman‐PCR for the quantification of tumour cell invasion into omentum. Genomic DNA from human OVCAR8 cells and mouse omentum (100 pg) were mixed at the indicated ratios and the signal for human DNA (hAlu sequences) was determined. The plot shows a linear relationship between signal intensity and the amount of human DNA. (G) Quantification by Taqman‐PCR of human DNA in omentum samples after incubation with spheroids generated from control and BCAM1‐overexpressing OVCAR8 cells as in panels A–E. The plot shows the data for *n* = 5–8 biological replicates as indicated by symbols. *p < .05; by *t*‐test. (H) Longitudinal ^18^F‐FDG PET/CT images of mice 28 days after i.p. injection of spheroids derived from OVCAR8 control cells and from two different clones of BCAM‐overexpressing OVCAR8 cells. Leftmost image: mouse not inoculated with tumour cells for comparison. The picture on the right shows large space‐occupying BCAM1‐8 tumour masses in the omentum (om) displacing the liver and other organs.

As demonstrated by light‐sheet microscopy of whole omentum specimens, BCAM1‐overexpressing OVCAR8‐OE cells invaded the omentum with an efficiency clearly exceeding that of OVCAR8 control cells (Figures 8A and B) by a median of approximately 10‐fold (Figure 8C). Milky spots are morphologically distinct areas in the omentum, which are characterised by a dense microvasculature, an abundance of immune cells and a discontinuous mesothelial layer, making milky spots preferred sites of early OC metastasis.^36^, ^57^, ^58^ Importantly, BCAM1‐overexpressing OVCAR8‐OE cells invaded not only milky spots but also areas distant to milky spots (arrows in Figure 8B), which is consistent with the observed BCAM‐dependent clearance of a mesothelial cell monolayer by tumour cells in vitro (Figure 7). Further analyses by multi‐photon microscopy confirmed that the invading tumour cells were homing to the sub‐mesothelial collagen areas of the omentum independent of the presence of milky spots (milky spot area in Figure 8D; area distant from milky spots in Figure 8E).

To allow for an independent quantification of tumour cells invading the omentum, we developed a Taqman‐PCR‐based assay for the specific detection of human tumour cell DNA. As shown in the validation experiment in Figure 8F, the assay faithfully reflected the number of tumour cells in a mixed population with mouse cells with a linear increase over a range of >10^4^. Using this assay, we found a >10‐fold stronger signal (approximately 4 Ct difference) for omentum exposed to BCAM1‐overexpressing OVCAR8 spheroids compared with spheroids from control cells (Figure 8G), confirming the microscopic enumeration of tumour cells above (Figure 8C).

Finally, we tested the metastatic potential of BCAM‐overexpressing OVCAR8 spheroids compared with spheroids derived from control cells in a mouse model. To this end, spheroids were injected i.p. and formation of tumour masses was observed for a period of 28 days by 18F‐FDG PET/CT imaging. As shown in Figure 8H, clearly elevated PET signals in the upper abdominal region harbouring the omentum were detected consistently with spheroids derived from two different clones of BCAM1‐overexpressing cells relative to corresponding control spheroids. Post‐mortem dissection confirmed massive colonisation of the omentum in mice inoculated with BCAM‐overexpressing spheroids (shown for BCAM1‐8 in Figure 8H), corroborating the observations made with the ex vivo model described above.

## 4 DISCUSSION

The present study provides new insights into the role of BCAM in the context of OC, which include the mechanism of BCAM shedding from tumour cells to release sBCAM, the hitherto unknown impact of sBCAM on metastasis‐related biological processes, a comparative functional analysis of sBCAM, BCAM1 and BCAM2 and the discovery of novel functions for BCAM with implications for peritoneal metastasis, including spheroid compaction/dispersion and mesothelial clearance. These results are summarised in the Graphical Abstract and discussed in detail below.

### 4.1 Role of ADAM10 in BCAM shedding

The origin and function of sBCAM in the tumo have not been addressed prior to the present study. BCAM in OC ascites is associated with a poor RFS (Figures 1C), but its source and function in OC progression were not investigated. Niiya and colleagues identified BCAM as a substrate of MMP14 in human epidermoid carcinoma A431 cells to produce a cleavage product of unknown function.^26^ We also detected MMP14 in the proteome of ascTU cells, but at low levels relative to several ADAM proteinases (Figure 2A). Consistent with these observations, analyses using proteinase inhibitors and MMP‐specific siRNAs clearly identified ADAM10 and ADAM17 as the proteinases majorly responsible for BCAM shedding, with ADAM10 playing a key role (Figures 2B–E).

The role of ADAM10 as a BCAM‐cleaving enzyme was confirmed using recombinant proteins (Figure 2F). MS‐based analysis identified cleavage sites in BCAM near the transmembrane domain and in the vicinity of the published MMP14 site (Figure 2G). One of these sites (site 3; Figure 2G) overlapped with a cleavage site found in BCAM from the culture supernatant of OC cells, and fitted the motifs of preferred amino acids at ADAM10 cleavage sites defined in previous studies^52^, ^53^ (Figure S12). These findings indicate that ADAM10 cleaves BCAM close to its insertion point in the plasma membrane to produce a soluble form (sBCAM) released into the TME. This conclusion was confirmed by immunoblotting identifying sBCAM rather than the longer membrane‐associated forms in OC ascites (Figure 1A). Based on these results, we analysed the function of sBCAM in comparison with BCAM1 and BCAM2 in metastasis‐related biological processes as discussed in the following sections.

### 4.2 Metastasis‐related functions of BCAM

Literature reports on the role of BCAM in cancer progression do not provide a consistent picture describing both tumour‐promoting and suppressive functions and clinical associations (see *Background*; Figure S1). As discussed in the following, these seemingly contradictory findings may be due to tumour‐entity‐related differences in the biology of tumour progression, which also have implications for interpreting the results of the present study.

In most cancer types, metastatic spread via blood or lymphatic vessels is mediated by migrating single tumour cells or groups of cells.^59^, ^60^ Components of the ECM, including laminins, are instrumental in processes involved in hematogenic and lymphogenic spreading, such as intravasation, extravasation and tissue invasion by cancer cells. LN‐511 is one of the most potent adhesive and migration‐promoting matrix components.^61^ It promotes integrin‐dependent tumour cell migration and invasion and exerts its effects partly via autocrine stimulation,^61^, ^62^, ^63^ which has been described for the promotion of breast cancer metastasis.^64^, ^65^ The inhibitory effect of LAMA5‐integrin signaling^5^ may therefore provide an explanation why BCAM expression is not associated with the short survival of these cancer entities (Figure S1B).

This contrasts with OC, where transcoelomic dissemination of cancer cells, particularly via spheroids, is the main route of metastasis.^23^, ^66^, ^67^, ^68^ Laminin‐integrin interactions have been reported to play a crucial role in compacting tumour cells in spheroids. For example, laminin networks mimicking a vasculogenic environment were found to be integral to the extracellular architecture and thereby the formation of melanoma spheroids in vitro.^69^ Likewise, ECM‐triggered ITGB1 signalling, including the addition of exogenous laminin (from Engelbreth–Holm–Swarm murine sarcoma basement membrane), promoted the formation and adhesion of OC spheroids.^70^, ^71^

Our own data points to context‐dependent functions for BCAM in OC cells differing in their potential impact on metastasis formation. On the one hand, BCAM inhibits the adhesion of single OC cells to LN‐511 (presumably by competing for integrin binding), which may have an inhibitory impact on metastasis, consistent with studies of gastrointestinal and bladder carcinoma cells.^5^, ^7^ However, BCAM does not inhibit adhesion to COL1 (Figures S6E–G), which is relevant given the crucial role of collagen in OC metastasis.^48^, ^50^, ^72^ On the other hand, spheroids are rich in LAMA5, (Table 2; Figures 3 and S13), which presumably provides a scaffold for OC cell adhesion via integrin binding. This interaction is prone to perturbation by BCAM,^5^ reducing the compactness of spheroids (Figure 5), promoting their dispersion in a 3D collagen matrix (Figure 6) and the clearance of mesothelial cells at spheroid attachment sites (Figure 7), which in turn likely enhances the seeding of metastatic colonies. Thus, the final consequences of the interaction of BCAM and LAMA5 and the ensuing inhibition of laminin‐dependent adhesion, depend on the precise scenario considered.

Milky spots are the preferred sites of early OC metastasis due to their discontinuous mesothelium,^36^, ^57^, ^58^ However, cancer cell invasion into serous membranes also occurs outside milky spots, where the mesothelial layer needs to be disrupted by the adhering tumour cells to initiate invasion, and this mode of metastatic seeding increases with disease progression. Our data obtained with explanted omentum suggest that BCAM promotes invasion into areas outside of milky spots, without detectable effects on invasion into milky spots (Figure 8B). This is in perfect agreement with our finding that tumour‐cell‐mediated clearance of a mesothelial cell monolayer is promoted by BCAM (Figure 7). Different mechanisms mediating disruption of the mesothelium by OC cells have been proposed, including mesothelial senescence^73^ and killing by secreted factors^74^ as well as myosin‐driven mechanical force exerted by tumour cells.^75^ In view of these complexities, the molecular mechanism(s) underlying BCAM‐induced mesothelial clearance have to remain the subject of future investigations.

### 4.3 Molecular basis of LAMA5‐dependent functions of BCAM

The full‐length BCAM isoform, BCAM1, is a transmembrane receptor for LAMA5 with an intracellular domain with potential signal transducing functions. It has been reported that overexpression of BCAM in NIH3T3 fibroblasts leads to F‐actin rearrangement via increased Erk phosphorylation, increased RhoA and decreased Rac1 activity, but the relevance of these findings in an endogenous context remains unclear. BCAM1 has also been shown to be phosphorylated by glycogen synthase kinase 3β, casein kinase II and PKA at serines 596, 598 and 621, respectively and the phosphorylation state of BCAM might be a critical factor for adhesion of erythrocytes to LAMA5 in sickle cell anemia.^76^ In tumour cells, BCAM‐mediated signal transduction has not been investigated to date.

Our data clearly suggest that BCAM exerts its biological functions in OC described in the present study not as a signalling receptor. This conclusion is based on the observations that BCAM1, BCAM2 and Fc‐BCAM had identical effects on OC cell adhesion, migration, motility and spheroid formation (Figures 1C, 1, 4 and S8). BCAM2 lacks most of the cytoplasmic domain, presumably impairing its signalling potential. Taken together with the data obtained with Fc‐BCAM, a function for BCAM as a signalling receptor in the context of the biological effects observed in the present study can be ruled out.

It is therefore more likely that BCAM acts by competing with another ligand for a signalling receptor or by interacting as a ligand or auxiliary protein with another receptor. BCAM has been reported to compete with β1‐containing integrins for laminin binding.^5^, ^16^ Our data strongly support the conclusion that a similar mechanism applies to the BCAM‐mediated inhibition of LN‐511‐dependent OC cell adhesion as well as spheroid compaction and dispersion. Thus, the effect of BCAM was counteracted by an integrin β1 activating antibody or by the addition of excess LN‐511 (Figures 5A–F and 6C and D). Furthermore, an integrin β1 blocking antibody had a similar effect as BCAM (Figures 5G and H). These observations strongly suggest that BCAM acts as a non‐signalling decoy receptor blocking the interaction of LAMA5 with β1‐containing integrins to diminish the attachment of single tumour cells to a matrix on the one hand, and to decrease the compactness of spheroids and enhance their dispersion at attachment sites on the other.

Soluble BCAM (as recombinant Fc‐BCAM) affects tumour cells in culture at a concentration corresponding to the highest sBCAM levels in ascites (Figures 1B, D and E), strongly supporting a role in the OC TME. Our data also indicate that Fc‐BCAM and overexpressed BCAM1 or BCAM2 are functionally similar (Figures 1D and E), raising the question as to whether the effect of membrane‐bound BCAM depends on its shedding. While cleavage of overexpressed BCAM is likely to contribute to the observed functions, kinetic experiments suggest that membrane‐bound forms play a role (Figure S7). Thus, cells overexpressing BCAM1 or BCAM2 showed clearly decreased adhesion to LN‐511 within less than 20 min after plating the cells, which is unlikely to suffice for the accumulation of a functionally relevant level of sBCAM. It thus appears that both membrane‐bound and soluble BCAM figure in modulating OC cell functions, consistent with the proposed role as a competing ligand. BCAM shedding nevertheless may be of particular importance to increase the pool of sBCAM in the TME. This is suggested by the observation that tumour‐associated host cells also express elevated levels of BCAM and/or BCAM‐cleaving proteases. This is documented in Figure S17, showing high expression of *BCAM* in mesothelial cells, *ADAM10* and *ADAM17* in CAF and *MMP14* in tumor‐assocaited macrophages (TAM) and carcinoma‐associated fibroblasts (CAF).

BCAM has also been identified as a ligand for integrin α4/β1 on leukocytes in murine glomerulonephritis,^12^ but an impact on signal transduction has not been investigated. Moreover, integrin α4 is selectively expressed in hematopoietic cells, and its expression is barely detectable in OC cells (TPM < 1; Figure S16), suggesting a minor role, if any, as a signalling receptor bound by BCAM. It is, however, possible that other unidentified receptor(s) exist that are activated by BCAM or co‐regulated by BCAM.

### 4.4 Translational perspectives

The association of an unfavourable clinical outcome with both *BCAM* RNA expression (Figure S1C) and ascites levels of sBCAM (Figure 1C), in conjunction with its pro‐metastatic function uncovered in the present study, suggests that BCAM may represent a novel therapeutic target in OC. Although such considerations remain hypothetical at present, several options can be devised and tested in future studies. A promising approach appears to be to prevent BCAM from its interaction with LAMA5. In this context, it is noteworthy that *KRAS*‐mutated colon carcinoma cells express high levels of BCAM and efficiently form hepatic metastases in mice, which is inhibited by BCAM‐mimetic peptides blocking LAMA5–BCAM interaction.^6^ Alternatively, it could be envisaged to develop molecules targeting BCAM to block its interaction interface, which is likely to result in lesser side‐effects than blocking LAMA5, which is essential for numerous crucial physiological functions.^77^ In this scenario, the development of small molecule drugs, particularly PROTACs,^78^ may represent a successful strategy. Furthermore, BCAM is a potential candidate for targeted immunotherapies, which could address both membrane‐bound and soluble BCAM. Preventing the production of sBCAM by blocking ADAM10 and/or other relevant metalloproteinases may also represent an option, but is less likely to succeed in view of the failure of marimastat in clinical trials, including ovarian cancer.^79^, ^80^ Even though BCAM‐directed approaches may not be suitable for the treatment of established metastatic lesions, they could provide invaluable tools to prevent de novo metastasis formation in an adjuvant scenario.

## 5 CONCLUSIONS

The role of BCAM in cancer progression is controversial and has not been addressed for OC to date, including the origin and potential function of soluble BCAM abundant in the OC microenvironment. In the present study we show that BCAM negatively regulates the compactness of LAMA5‐rich tumour cell spheroids, and consequently triggers the dispersion of spheroids in a collagen matrix, facilitates the clearance of mesothelial cells at spheroid attachment sites and promotes the trans‐mesothelial invasion of tumour cell spheroids into omental tissue.

We also present compelling evidence suggesting that BCAM acts as a decoy rather than a signalling receptor to modulate metastasis‐related functions on OC cells. This conclusion is supported by the observation that full‐length BCAM1 and the truncated isoform BCAM2 lacking most of the cytoplasmic domain with potential signalling function had the same effect in different biological assays. Furthermore, we have identified ADAM10 as a major BCAM sheddase produced by OC cells and identified proteolytic cleavage sites yielding a soluble BCAM isoform exclusively composed of the extracellular domain. Recombinant soluble BCAM mimicking this isoform had the same effect as the membrane‐bound BCAM proteins.

Mechanistically, all BCAM forms interfered with interaction of LAMA5 and integrin‐β1. On the one hand, this results in decreased adhesion of single cells to a LN‐511 matrix, potentially representing an anti‐metastatic function. However, according to previous studies adhesion of OC cells to collagen rather than laminin drives peritoneal dissemination.^45^, ^46^, ^47^, ^48^, ^49^, ^50^ Importantly, BCAM has no detectable effect on adhesion to COL1, suggesting that the observed inhibitory effect of BCAM on the single‐cell adhesion to LN‐511 is of minor significance in this context. On the other hand, BCAM loosens the structure of spheroids, where LAMA5–integrin β1 interaction is essential to maximise compaction, thereby promoting the dispersion of cancer cell spheroids at target sites, which in turn is likely to contribute to peritoneal metastatic spread. This conclusion is consistent with the observed colonisation‐enhancing effect of BCAM in both explanted omentum and a mouse model, as well as the highly significant association of BCAM with a poor clinical outcome of OC. Our data not only provide new mechanistic insights into OC biology, but may also pave the way for therapeutic strategies impacting peritoneal metastasis formation.

## CONFLICT OF INTEREST

The authors declare no competing financial interests.

## ETHICS STATEMENT

All experiments were carried out with informed consent by the patients and approval by the ethics committee of Marburg University (205/10). All patients have agreed in writing to the publication of pseudonymized data derived from clinical materials.

## Supporting information

Supporting Information

Supporting Information

Supporting Information

Supporting Information
