# Supplementary material for: Basal cell adhesion molecule promotes metastasis‐associated processes in ovarian cancer
Source: Clin Transl Med. 2023 Jan 16;13(1):e1176. doi: 10.1002/ctm2.1176 (PMC9842900; doi:10.1002/ctm2.1176)
Supplement: Supplementary file 1 — Supporting Information [file CTM2-13-e1176-s002.pdf]

SUPPLEMENTAL FIGURES

Basal cell adhesion molecule (BCAM) promotes metastasis-associated processes in ovarian cancer

Suresh Sivakumar, Sonja Lieber, Damiano Librizzi, Corinna Keber, Leah Sommerfeld, Florian Finkernagel, Katrin Roth, Silke Reinartz, Jörg Bartsch, Johannes Graumann, Sabine Müller-Brüsselbach and Rolf Müller

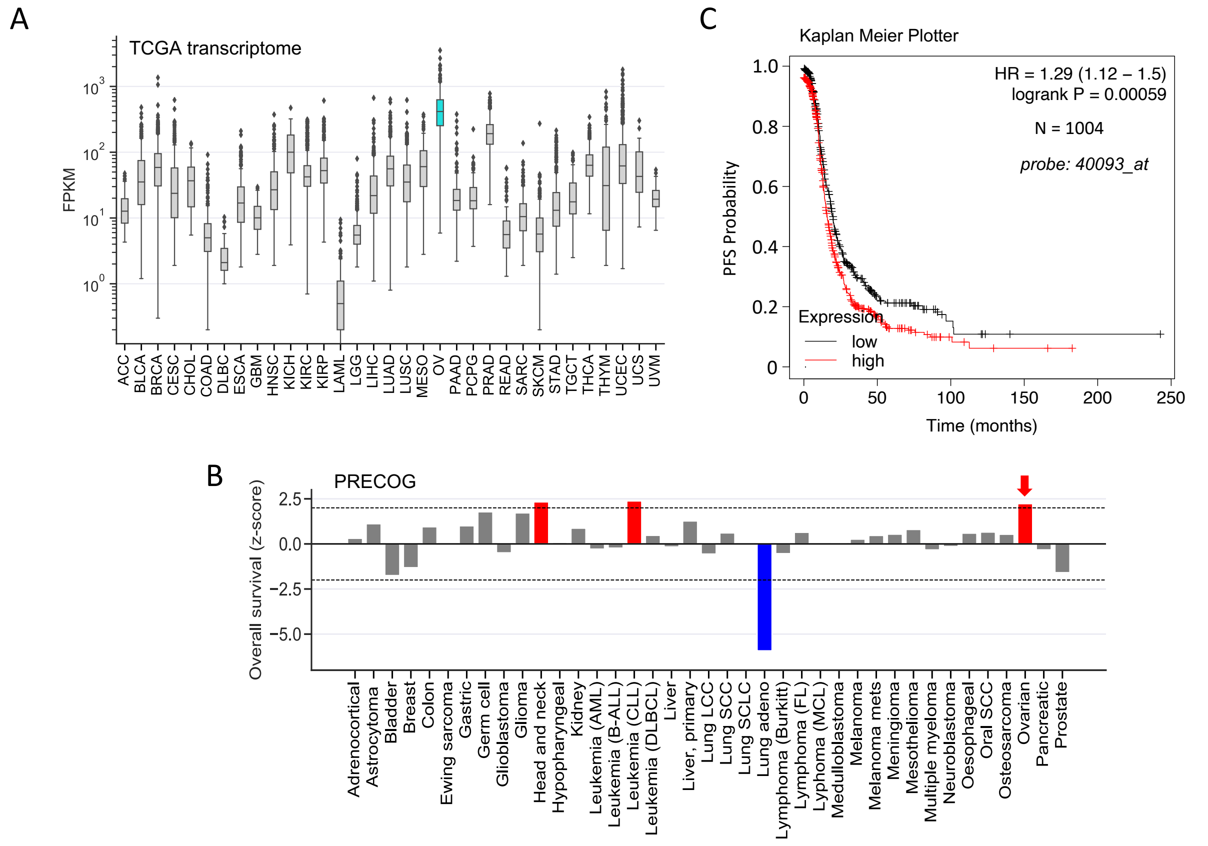

Figure S1: Expression of *BCAM* mRNA and association with overall survival (OS) in different cancer entities and assoc.

(A) Data were retrieved from the TCGA transcriptome dataset (TCGA Research Network, 2011). Boxplots show medians (horizontal line in boxes), upper and lower quartiles (box), and range (whiskers). **(B)** Data retrieved from the PRECOG dataset (Gentles et al., 2015). Positive and negative z-scores indicate HR>1 and HR<1, respectively. A z-score of |1.96| corresponds to a logrank p-value of 0.05. Red: significant association with a short OS; blue: significant association with a long OS. **(C)** Association of *BCAM* RNA expression with relapse-free survival (RFS) of OC. The plot was generated by the Kaplan-Meier Plotter online tool (Gyorffy et al., 2012) using the following settings: Jet set best probe; auto-select best cutoff; histology: serous; all other parameters: default).

ACC: Adrenocortical carcinoma; BLCA: Bladder Urothelial Carcinoma; BRCA: Breast invasive carcinoma; CESC: Cervical squamous cell carcinoma and endocervical adenocarcinoma; CHOL: Cholangiocarcinoma; COAD: Colon adenocarcinoma; DLBC: Lymphoid Neoplasm Diffuse Large B-cell Lymphoma; ESCA: Esophageal carcinoma; GBM: Glioblastoma multiforme; HNSC: Head and Neck squamous cell carcinoma; KICH: Kidney Chromophobe; KIRC: Kidney renal clear cell carcinoma; KIRP: Kidney renal papillary cell carcinoma; LAML: Acute Myeloid Leukemia; LGG: Brain Lower Grade Glioma; LIHC: Liver hepatocellular carcinoma; LUAD: Lung adenocarcinoma; LUSC: Lung squamous cell carcinoma; MESO: Mesothelioma; OV: Ovarian serous cystadenocarcinoma; PAAD: Pancreatic adenocarcinoma; PCPG: Pheochromocytoma and Paraganglioma; PRAD: Prostate adenocarcinoma; READ: Rectum adenocarcinoma; SARC: Sarcoma; SKCM: Skin Cutaneous Melanoma; STAD: Stomach adenocarcinoma; TGCT: Testicular Germ Cell Tumors; THCA: Thyroid carcinoma; THYM: Thymoma; UCEC: Uterine Corpus Endometrial Carcinoma; UCS: Uterine Carcinosarcoma; UVM: Uveal Melanoma.´

References:

The Cancer Genome Atlas (TCGA) Research Network. Integrated genomic analyses of ovarian carcinoma. Nature 474, 609–615 (2011).

Gentles, A.J. et al. The prognostic landscape of genes and infiltrating immune cells across human cancers. Nat Med 21, 938–945 (2015).

Gyorffy, B., Lanczky, A. & Szallasi, Z. Implementing an online tool for genome-wide validation of survival-associated biomarkers in ovarian-cancer using microarray data from 1287 patients. Endocr Relat Cancer 19, 197–208 (2012). Website: http://kmplot.com/analysis/index.php?p=service&cancer=ovar

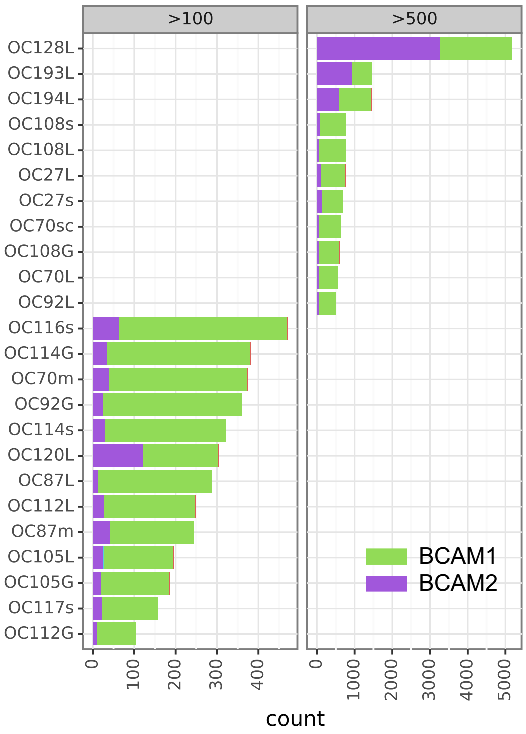

Figure S2: Expression of *BCAM1* versus *BCAM2* mRNA in tumor cells from ascites.

The plot is based on exon spanning reads from RNA-Seq data published in Sommerfeld et al. (2021).

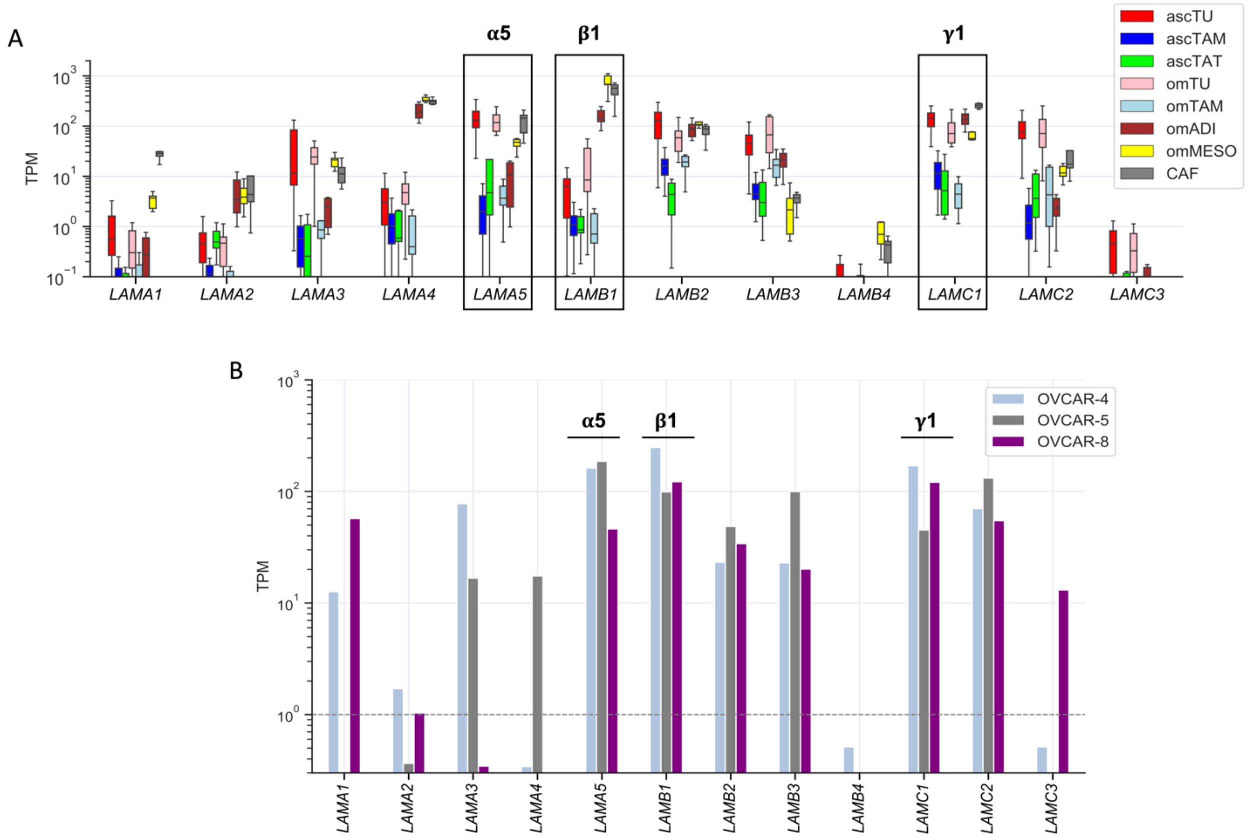

Figure S3: Expression of genes coding for laminin family members in cells of the OC TME and in OVCAR cell lines based on RNA-Seq data.

(A) Expression in tumor and host cells in OC ascites and omental metastases. The plot is based on RNA-Seq data retrieved from Sommerfeld et al. (2021). asc: ascites-derived; om: isolated from omental metastases; TU: tumor cells; TAM: tumor-associated macrophages; TAT: tumor-associated T cells; ADI: adipocytes; MESO: mesothelial cells; CAF: fibroblasts. Boxplots show medians (horizontal line in boxes), upper and lower quartiles (box), and range (whiskers).

(B) Expression in OVCAR4, OVCAR5 and OVCAR8 cells. Data for were generated by RNA-Seq as described in Sommerfeld et al. (2021).

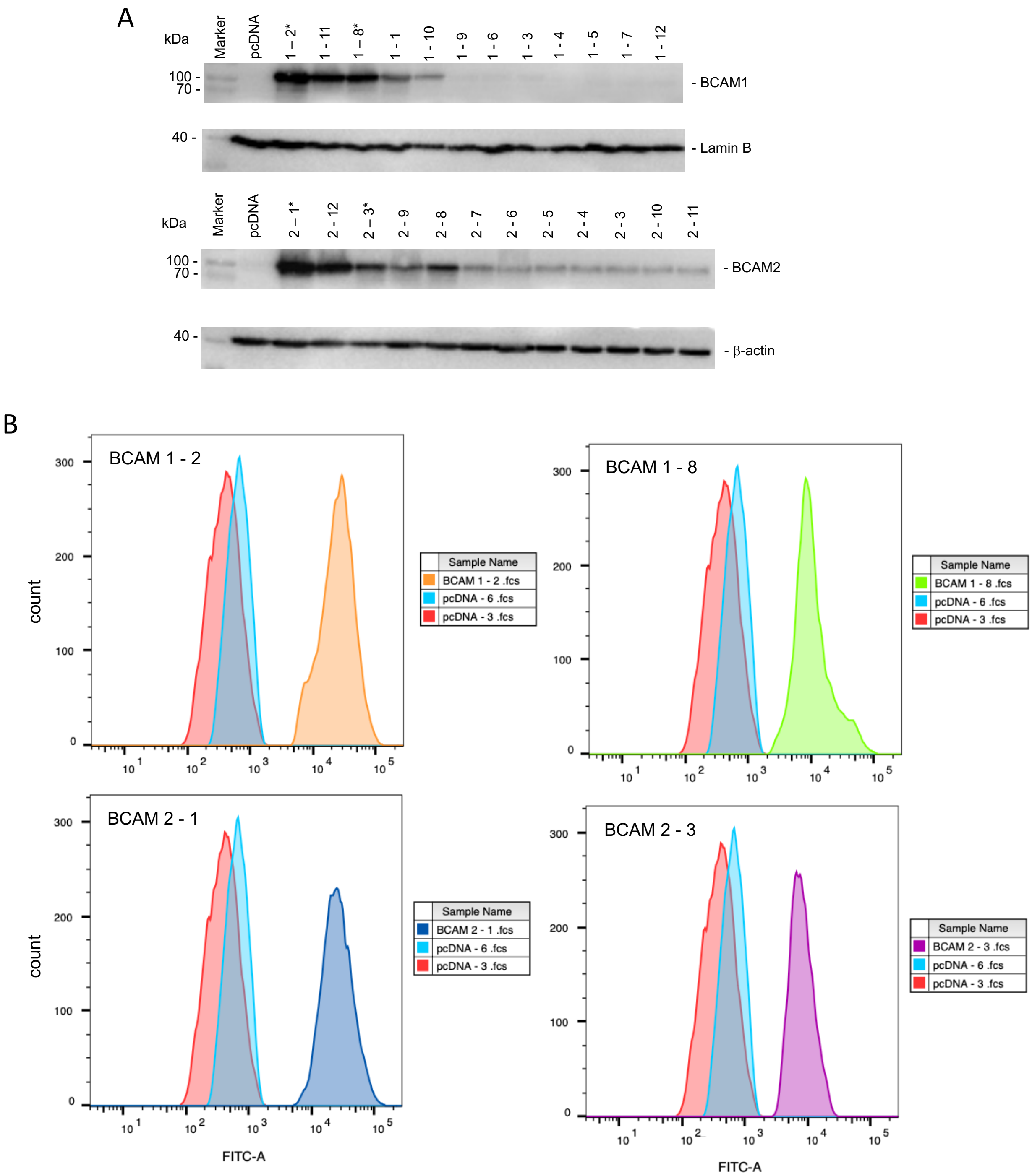

Figure S4: BCAM expression in OVCAR8 clones stably expressing ectopic BCAM1 or BCAM2.

(A) Immunoblot analysis of of BCAM. β-actin was included as loading control. *clones used for functional experiments in the present study.

(B) Flow cytometric analysis of BCAM surface expression in OVCAR8 clones stably expressing ectopic BCAM1 or BCAM2. Experimental details were as described in the main text.

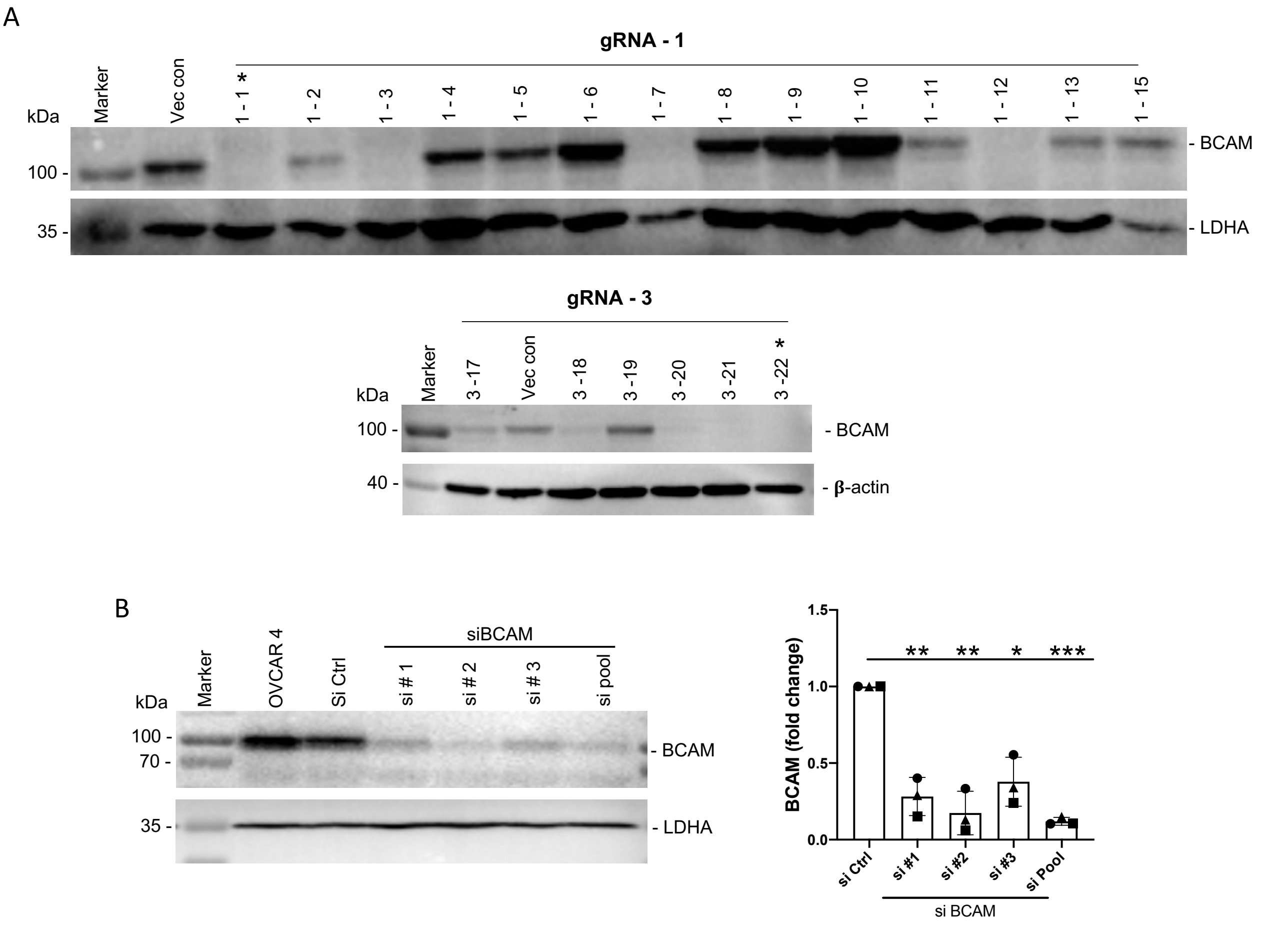

Figure S5: Immunoblot verification of *BCAM* gene disruption and *BCAM* targeting siRNA.

(A) Immunoblot verification of CRISPR-Cas9-mediated BCAM disruption in individual OVCAR8 clones. β-actin and LDHA were included as loading controls. Vec con: empty vector control. *clones used for functional experiments in the present study.

(B) Immunoblot analysis of siRNA-mediated inhibition of BCAM in OVCAR4 cells. Different siRNAs (si #1, #2, #3) were tested both individually and pooled. si Ctrl: unrelated control siRNA. α-tubulin and LDHA were included as loading controls.

Experimental details were as described in the main text.

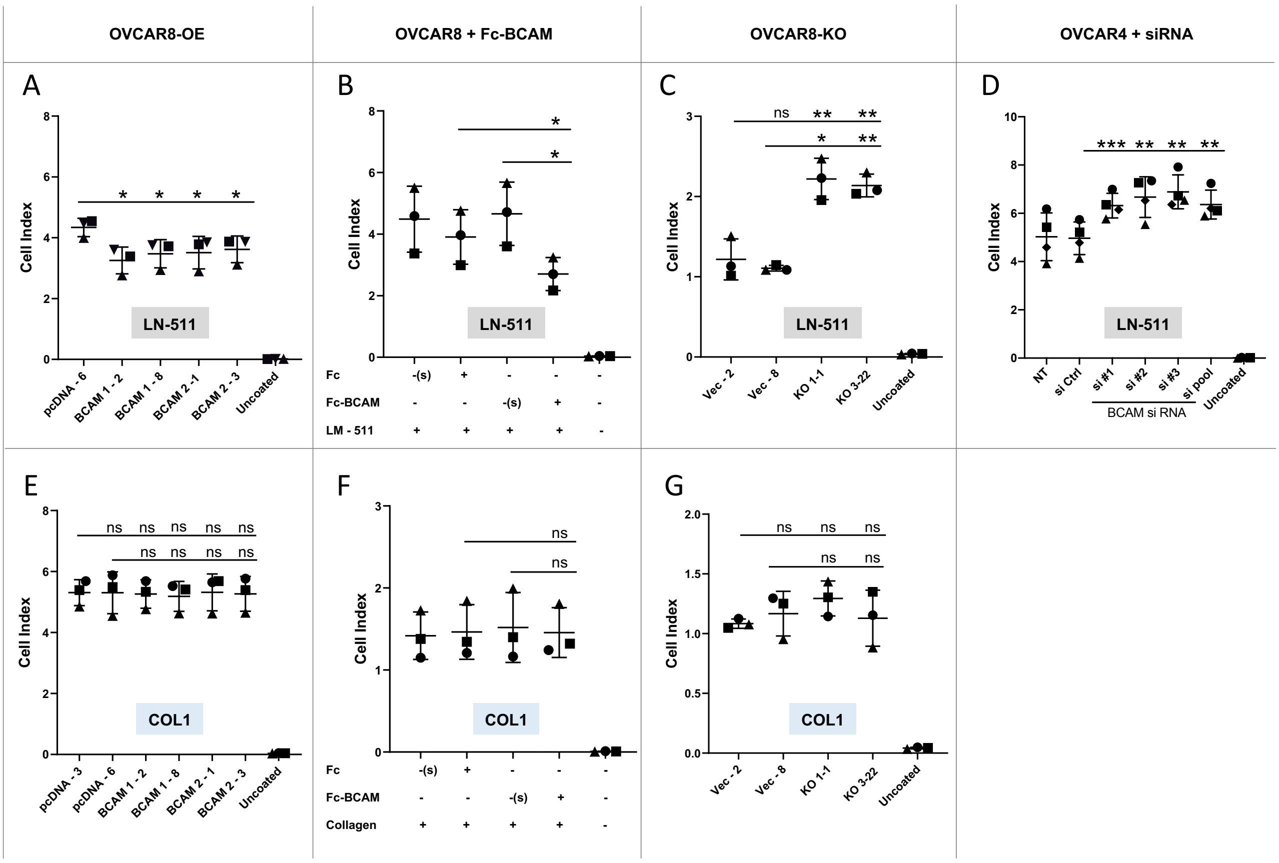

Figure S6. Effect of BCAM on OC cell adhesion to LN-511 and COL1.

Cells were seeded on non-adhesive microplates coated with LN-511 (panels A-D) or COL1 (panels E-G) and cell adhesion was quantified by RTCA.

NOTE: *Panels A and B are identical to Fig. 1D. They are included here to allow for a better comparison*

(**A, E)** BCAM-overexpressing OVCAR8 cells (OVCAR8-OE). Clones stably transfected with BCAM1 or BCAM2 (Fig. S4) were compared to cells transduced with the empty expression vectors (pcDNA-3, pcDNA-6).

(B, F) OVCAR8 cells were analyzed in the presence of Fc-BCAM or negative control (Fc). (s): solvent for Fc or Fc-BCAM. The data are based on n=3 biological replicates. *p<0.05; **p<0.01; ***p<0.001; ns, not significant by unpaired t test.

(C, G) OVCAR8 cells with disrupted BCAM gene (OVCAR8-KO) compared to cells transduced with the empty vector (clones Vec-2, Vec-8).

(D) OVCAR4 cells transfected with control-siRNA, 3 different BCAM-siRNAs (#1, #2, #3) or pooled siRNAs (pool). NT: non-transfected cells. Uncoated: cells plated on uncoated dishes.

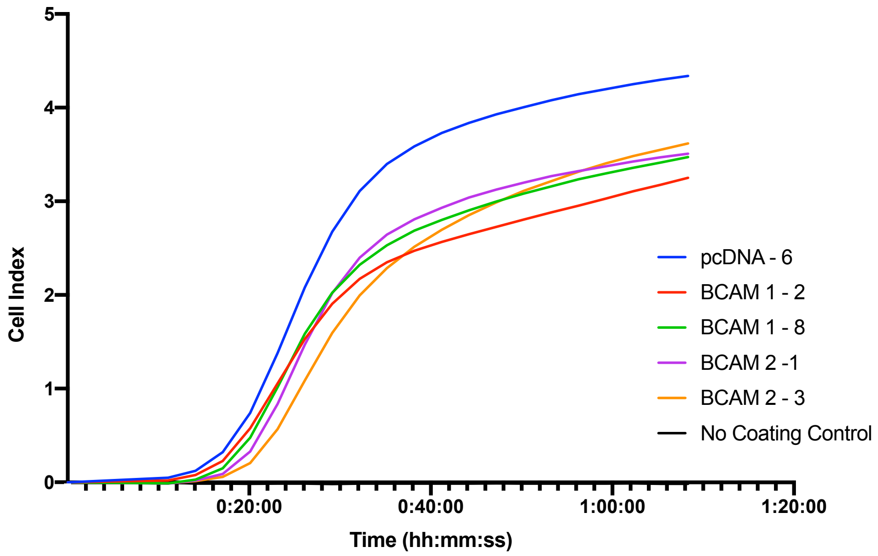

Figure S7. Kinetics of OVCAR8 cell adhesion to LN-511.

Cells were seeded on non-adhesive microplates coated with LN-511 (or uncoated control plates) and cell adhesion was quantified by RTCA as in Fig. S6. Clones overexpressing BCAM1 or BCAM2 were compared to cells transduced with the empty expression vector (pcDNA-6).

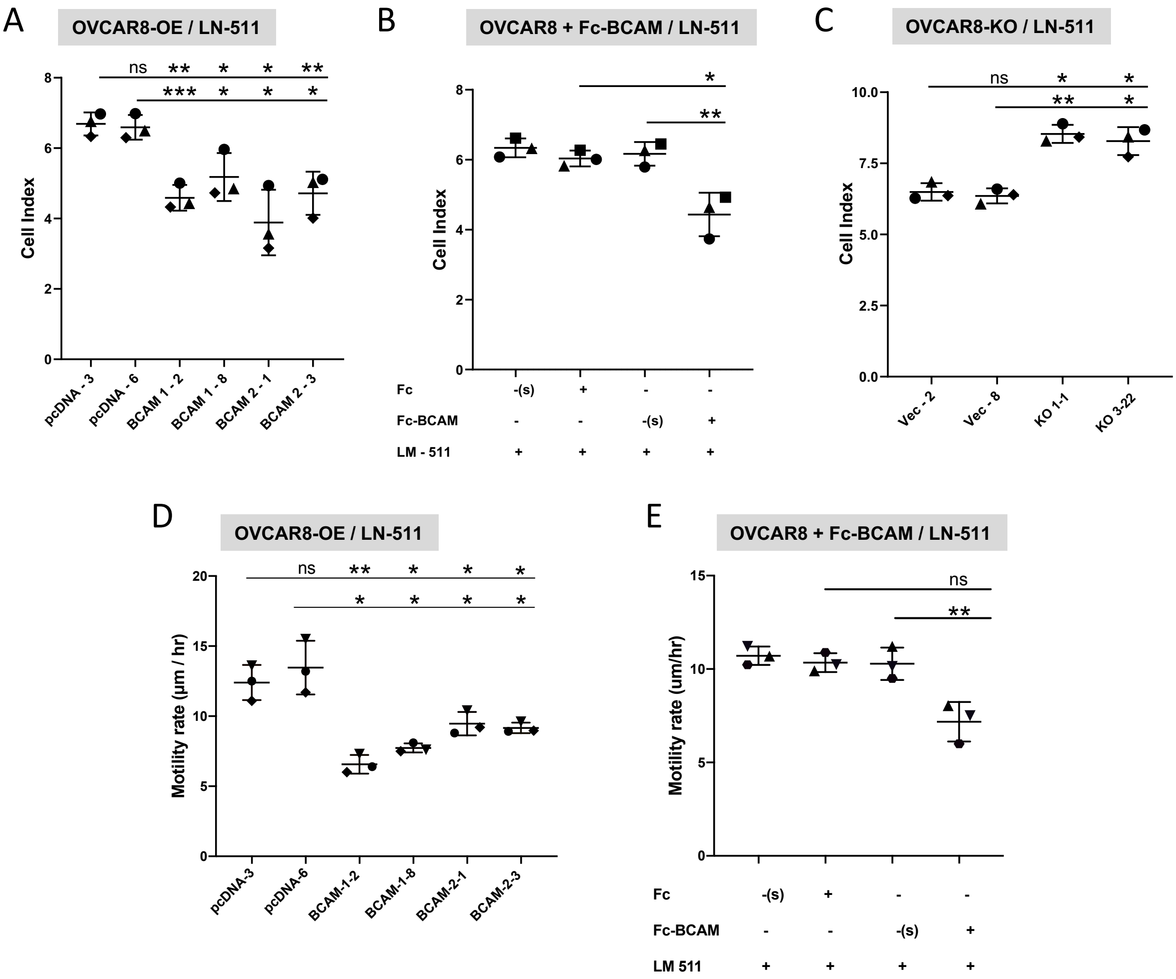

Figure S8. Effect of BCAM on two-dimensional OC cell migration and motility.

NOTE: *Panels A and B are identical to Fig. 1E. They are included her to allow for a better comparison.*

(A-C) Quantification of cell migration. Cells were seeded on transwell-chamber microplates coated with LN-511 and cell migration was quantified by RTCA.

(**A**) BCAM-overexpressing OVCAR8 cells (OVCAR8-OE; 2 BCAM1 and 2 BCAM2 clones) compared to cells transduced with the empty vector (2 clones).

(B) OVCAR8 cells analyzed in the presence of negative control (Fc) or Fc-BCAM and plated on LN-511. The data are based on n=3 biological replicates.

(C) OVCAR8 cells with disrupted BCAM (OVCAR8-KO) compared to cells transduced with the empty vector.

(D, E) Quantification of cell motility. The plots show quantifications of non-directional movement of OVCAR8 cells overexpressing BCAM1 or BCAM2 in panel D, or exposed to Fc or Fc-BCAM in panel E. Fluorescently labeled cells were observed for 12 hrs. Positions of cells were tracked to quantify cell motility. For each condition, trajectories were tracked in 5 different fields. “(s)” in panels C and E: solvent for Fc or Fc-BCAM. Data are shown for n=3 biological replicates in each panel. *p<0.05; **p<0.01; ***p<0.001; ns, not significant by unpaired t test.

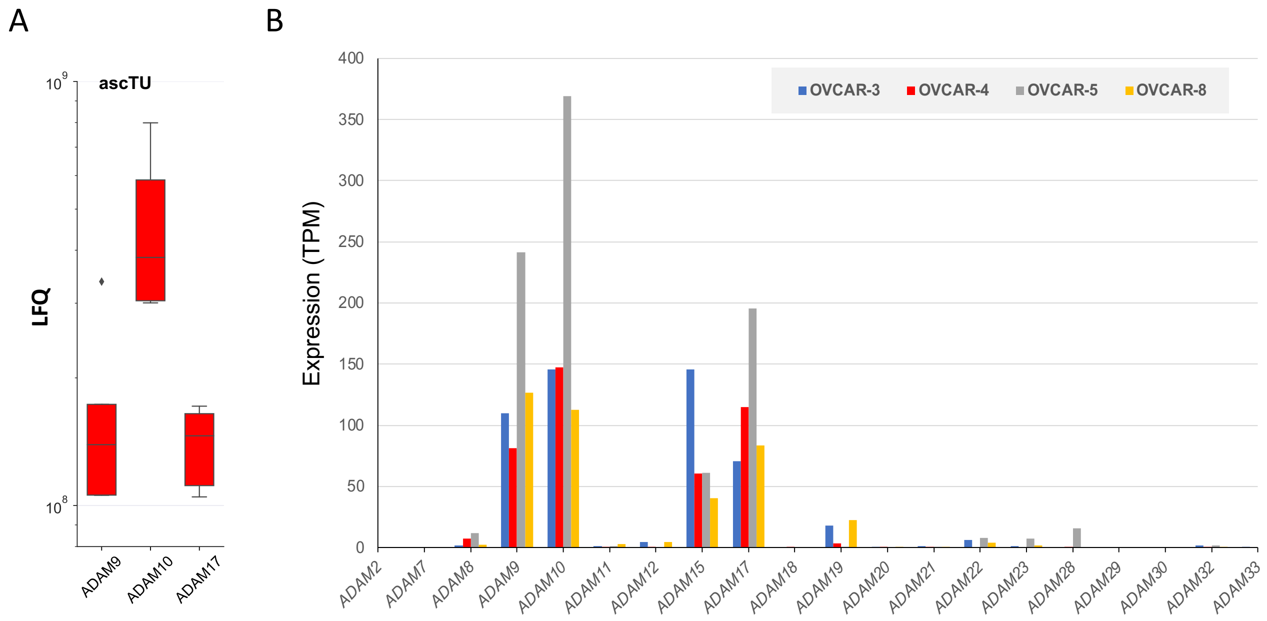

Figure S9: Release of ADAMs by asTU cells and expression of ADAM genes in OVCAR cell lines.

(A) Proteomic analysis of ADAM proteins in the secretome of ascTU cells (conditioned medium after short-term culture) as previously described (Worzfeld et al., 2018).

(B) Data for OVCAR4, OVCAR5 and OVCAR8 cells were generated by RNA-Seq as described in Sommerfeld et al. (2021). Data for OVCAR3 cells were retrieved from the literature (McCorkle et al., 2021) and included after re-processing of original data, and are therefore not directly comparable to the other datasets.

References:

Worzfeld, T., Finkernagel, F., Reinartz, S., Konzer, A., Adhikary, T., Nist, A., et al. Proteotranscriptomics Reveal Signaling Networks in the Ovarian Cancer Microenvironment. Mol Cell Proteomics 2018;17:270-289.

McCorkle JR, Gorski JW, Liu J, Riggs MB et al. Lapatinib and poziotinib overcome ABCB1-mediated paclitaxel resistance in ovarian cancer. PLoS One 2021;16(8):e0254205.

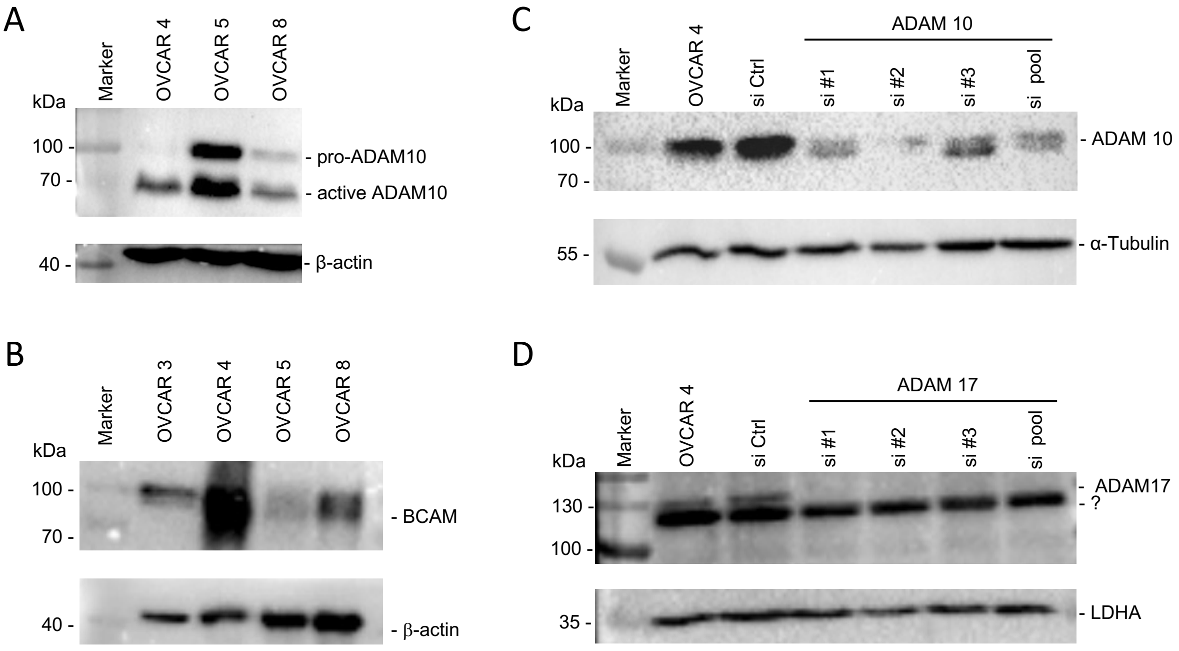

Figure S10: Immunoblot analysis of ADAM10, ADAM17 and BCAM in OVCAR cell lines.

(A) Immunoblot analysis of ADAM10 in OVCAR cell lines. β-actin was included as loading control

(B) Immunoblot analysis of BCAM in OVCAR cell lines. β-actin was included as loading control.

(C, D) Immunoblot analysis of siRNA-mediated inhibition of ADAM10 and ADAM17 in OVCAR4 cells. Different siRNAs (si #1, #2, #3) were tested both individually and pooled. siCtrl: unrelated control siRNA. α-tubulin and LDHA were included as loading controls.

Experimental details were as described in the main text.

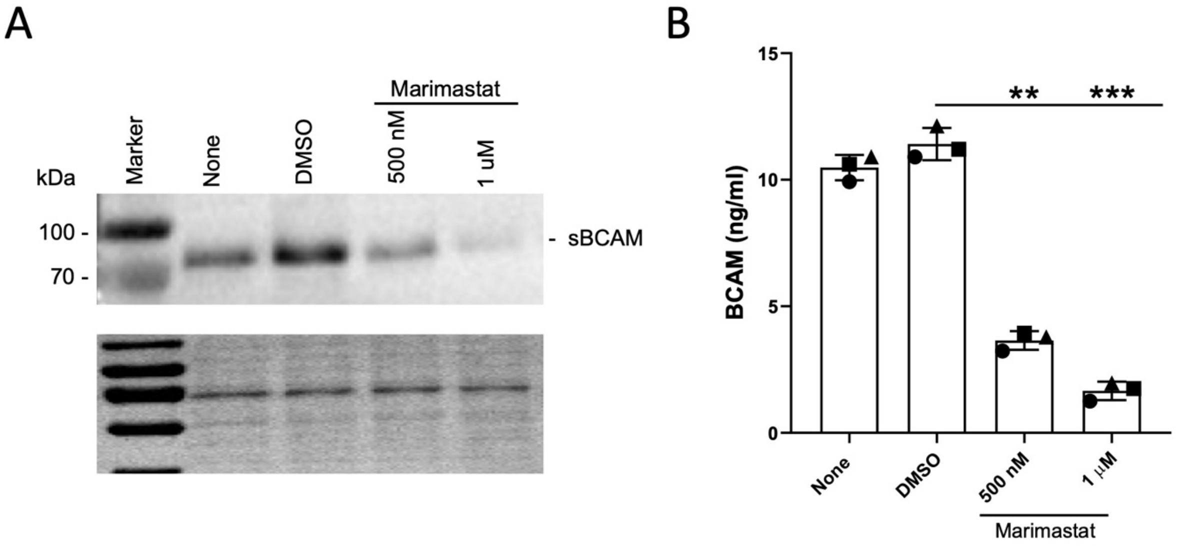

Figure S11: Effect of marimastat on sBCAM production.

(A) Immunoblot of medium from OVCAR4 cells cultured in the presence of different concentrations of marimastat for 24 hrs. The bottom panel shows the membrane after staining with the Pierce Reversible Total Protein Stain Kit as loading control. **(B)** Analysis of BCAM release by OVCAR4 cells treated as in panel A. Cell culture media were analyzed by ELISA 24 hrs after medium change. The plot shows the results of 3 independent experiments. *p<0.05; **p<0.01; ***p<0.001 by unpaired t test.

A

	enriched 1-2x	enriched >2x
**P5**	A G Q S	C H P Y
**P4**	A C E H I K Q R S V Y	T W
**P3**	C F K N P Q S V	A H M T Y
**P2**	E H L Q S	A C M
**P1**	C E F H K M	A P R Y
**P1'**	F I M Q R V	C H L W Y
**P2'**	A I K L Q R S T V Y	H M
**P3'**	A D L M Q R S T Y	G W
**P4'**	A C E G H P Q R S T	D
**P5'**	A D G H L M N P Q Y	R

B

Site 1 (rec-ADAM10)

```
L S W S Q * L G G S P
  •   •     +   + • •
```

Site 2 (rec-ADAM10)

```
S R D G I * S C E A S
• •               •
```

Site 3 (TU-sec & rec-ADAM10)

```
G I S C E * A S N P H
• • • + •     •   • •
```

Site 4 (TU-sec, close to TM-domain)

```
G T V S P * Q T S Q A
• + • • +   • • • • •
```

Site 5 (TU-sec, next to TM-domain)

```
Q T S Q A * G V A V M
• + • • +     • •   •
```

Figure S12: Analysis of ADAM10 cleavage sites in BCAM protein. (A) Preferential amino acids around ADAM10 cleavage sites (between positions -5 and 5). The table integrates data from two published studies using unbiased screening approaches (Caescu et.al. 2009; Tucher et al., 2014) and distinguishes strongly enriched (>2x) and weakly enriched (1-2x) amino acids. **(B)** Matches of cleavage sites 1-5 (see Fig. 2G) with the consensus motif in panel A. +, match with strongly enriched amino acid; •, match with weakly enriched amino acid (as defined in panel A). Note that cleavage at sites 4 and 5 was not observed in the Fc-BCAM fusion protein, probably due to steric hindrance in the hinge region between BCAM and Fc tag.

Patient OC26

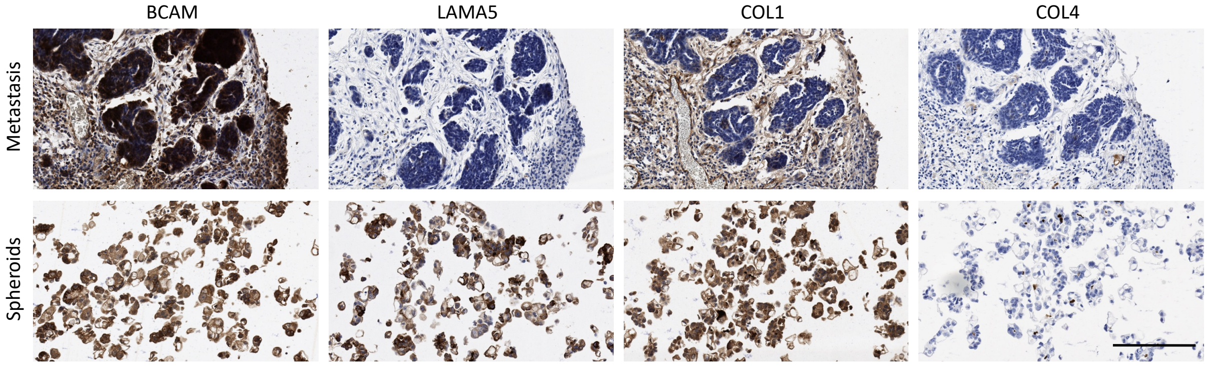

Patient OC84

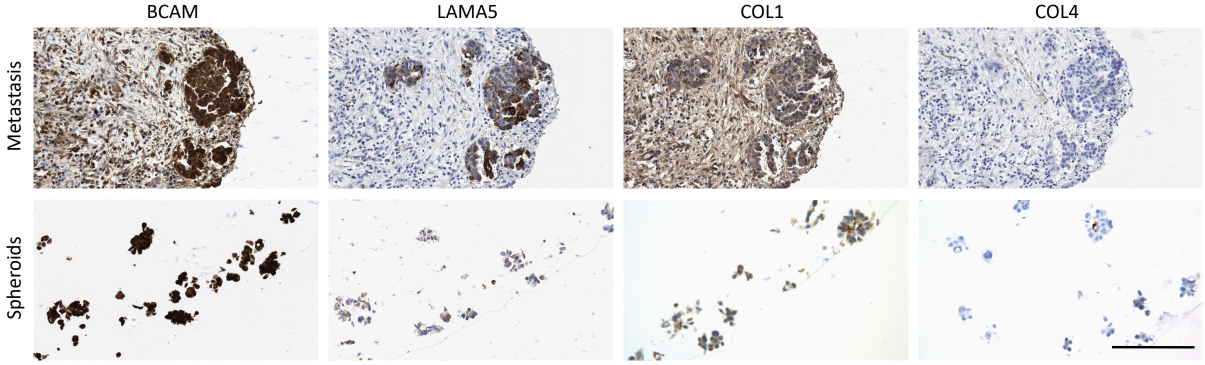

Patient OC122

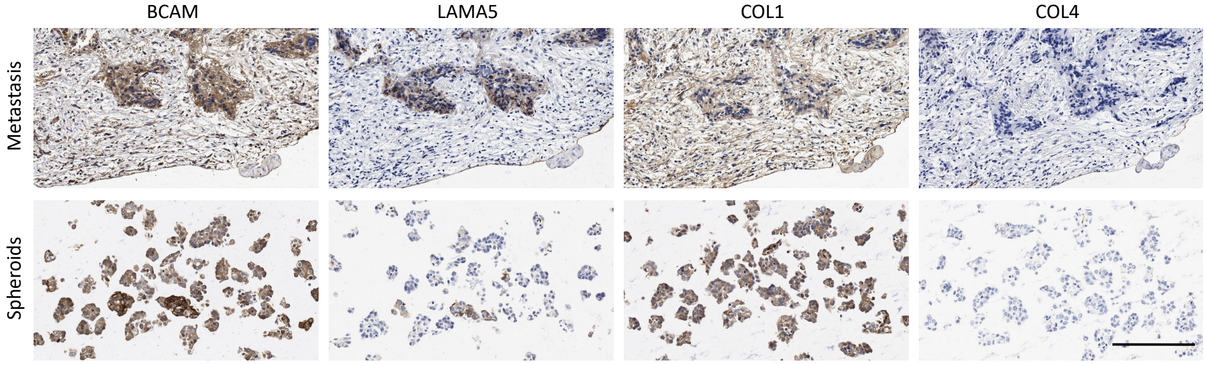

Figure S13: Immunohistochemical analysis of BCAM, LAMA5, COL1 and COL4 in matched samples of metastases and spheroids from ascites of 3 different OC patients.

Paraffin sections from metastases and spheroids form ascites were stained by immunohistochemistry as described in Materials and Methods. Quantifications of the images are shown in Table 2. Scale bar: 200 µm .

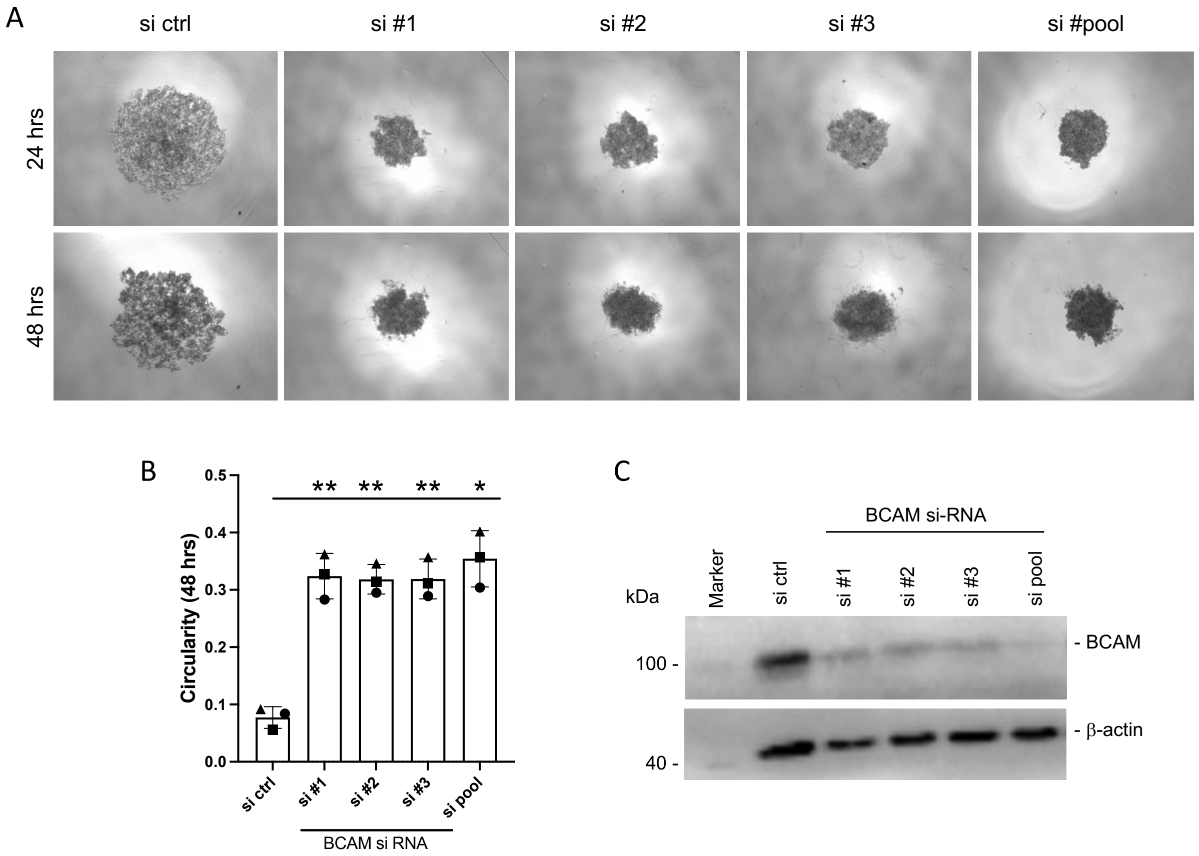

Figure S14: Impact of siRNA-mediated inhibition of BCAM expression on OVCAR3 spheroid formation.

(A) Morphology of spheroids derived from OVCAR3 cells transfected with control-siRNA (si ctrl), 3 different BCAM-siRNAs (#1, #2, #3) or pooled siRNAs (pool).

(B) Quantification of 3 independent experiments. *p<0.05; **p<0.01 by unpaired t test.

(C) Immunoblot analysis of siRNA-mediated inhibition of BCAM expression in OVCAR3 cells. Different siRNAs (si #1, #2, #3) were tested both individually and pooled. si ctrl: unrelated control siRNA. Experimental details were as described in the main text. β-actin was included as loading control

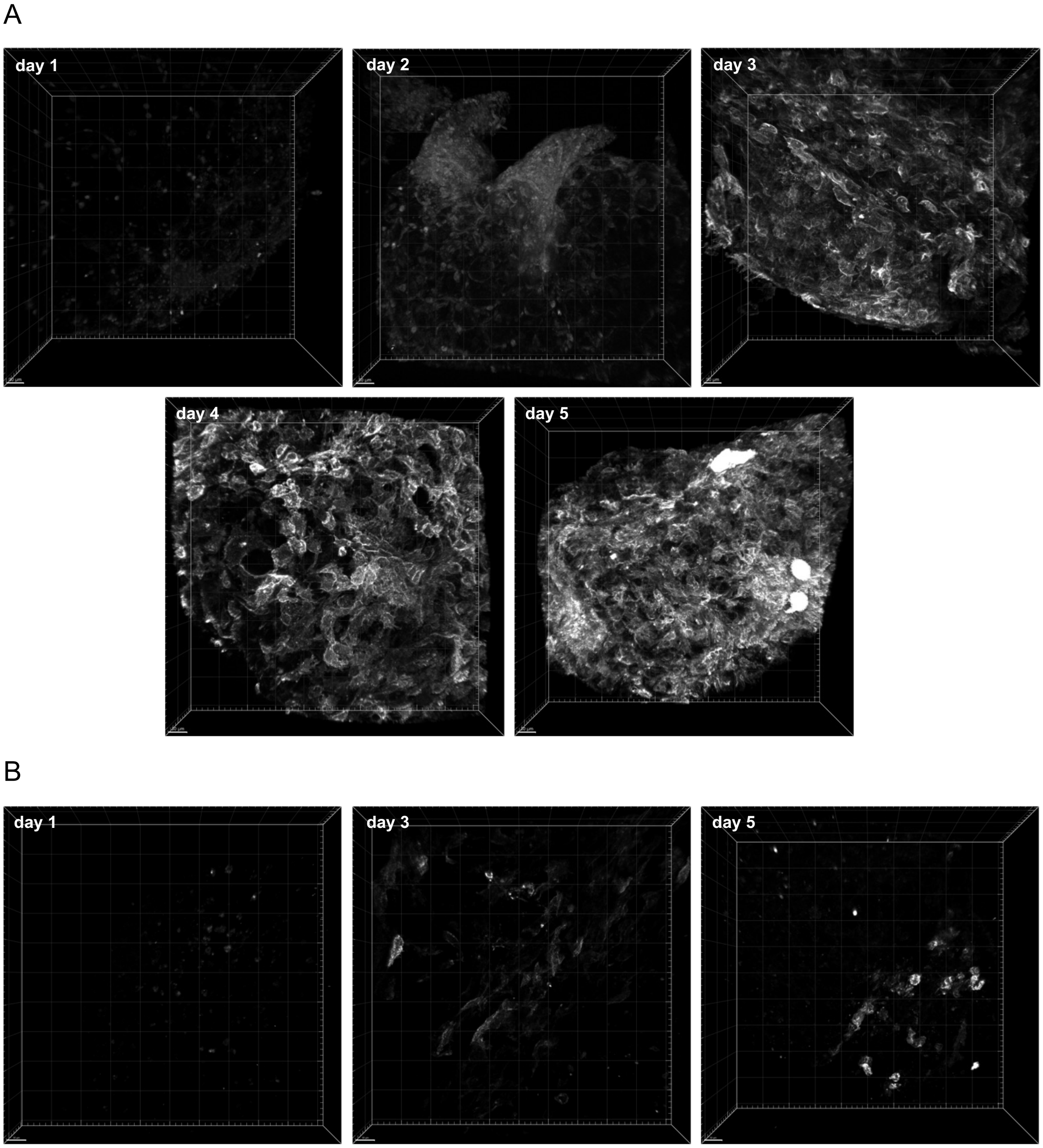

Figure S15: Expression of VCAM1 in mouse omentum at different times of culture *ex vivo* under different culture conditions.

(A) Culture *ex vivo* under standard culture conditions (hyperoxia, 5% regular FCS).

(B) Culture *ex vivo* under adapted culture conditions (hypoxia, delipidized FCS).

VCAM1 (CD106) was visualized (white) by immunostaining followed by multiphoton microscopy as described in Materials and Methods.

Scale bar: 30 µM.

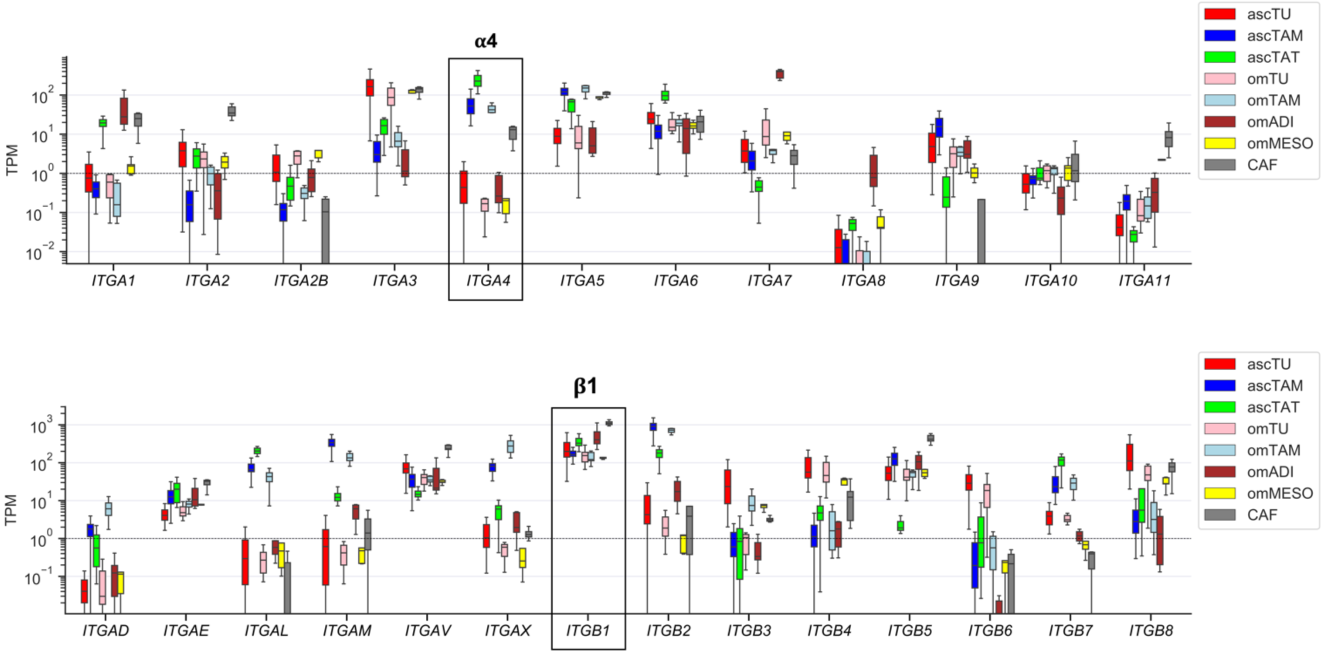

Figure S16: Expression of genes coding for integrin subunits in tumor and host cells of the OC TME.

The plot is based on RNA-Seq data from Sommerfeld et al. (2021)

Framed: integrin α4/β1 (BCAM receptor) subunits.

asc: ascites-derived; om: isolated from omental metastases; TU: tumor cells; TAM: tumor-associated macrophages; TAT: tumor-associated T cells; ADI: adipocytes; MESO: mesothelial cells; CAF: fibroblasts.

Boxplots show medians (horizontal line in boxes), upper and lower quartiles (box), and range (whiskers).

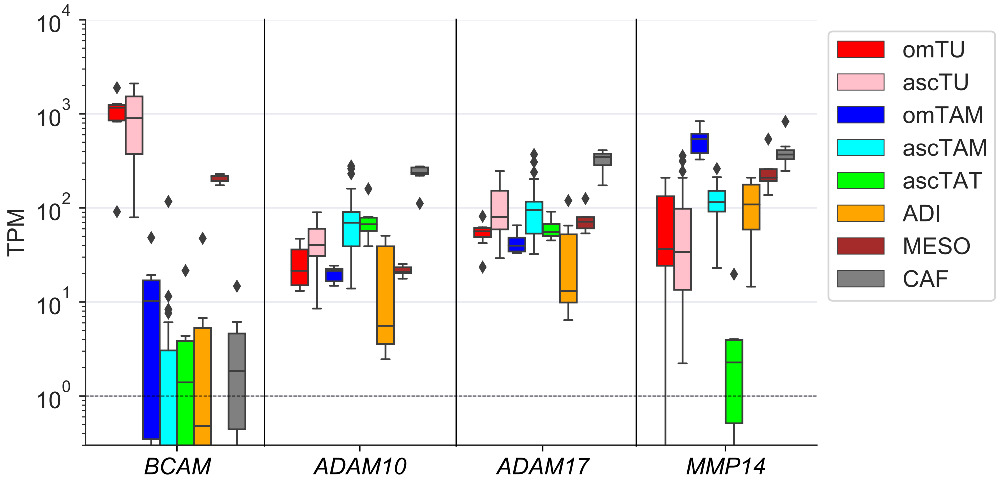

Figure S17: Expression of BCAM and metalloprotease genes in tumor and host cells of the OC TME.

The plot is based on RNA-Seq data from Sommerfeld et al. (2021)

asc: ascites-derived; om: isolated from omental metastases; TU: tumor cells; TAM: tumor-associated macrophages; TAT: tumor-associated T cells; ADI: adipocytes; MESO: mesothelial cells; CAF: fibroblasts.

The plot shows medians (horizontal line in boxes), upper and lower quartiles (box), and range (whiskers).
